# DNA methylation dynamics during intestinal stem cell differentiation reveals enhancers driving gene expression in the villus

**DOI:** 10.1186/gb-2013-14-5-r50

**Published:** 2013-05-28

**Authors:** Lucas TJ Kaaij, Marc van de Wetering, Fang Fang, Benjamin Decato, Antoine Molaro, Harmen JG van de Werken, Johan H van Es, Jurian Schuijers, Elzo de Wit, Wouter de Laat, Gregory J Hannon, Hans C Clevers, Andrew D Smith, René F Ketting

**Affiliations:** 1 Hubrecht Institute-KNAW & University Medical Centre Utrecht, Uppsalalaan 8, 3584 CT Utrecht, The Netherlands; 2 Howard Hughes Medical Institute, Cold Spring Harbor Laboratory, 1 Bungtown Road, Cold Spring Harbor, NY 11724, USA; 3 Molecular and Computational Biology, University of Southern California, Los Angeles, CA 90089, USA; 4 Institute for Molecular Biology, Ackermannweg 4, D-55128 Mainz, Germany

**Keywords:** Adult stem cells, Differentiation, DNA Methylation, Methylome, Enhancer, Tcf4

## Abstract

**Background:**

DNA methylation is of pivotal importance during development. Previous genome-wide studies identified numerous differentially methylated regions upon differentiation of stem cells, many of them associated with transcriptional start sites.

**Results:**

We present the first genome-wide, single-base-resolution view into DNA methylation dynamics during differentiation of a mammalian epithelial stem cell: the mouse small intestinal Lgr5+ stem cell. Very little change was observed at transcriptional start sites and our data suggest that differentiation-related genes are already primed for expression in the stem cell. Genome-wide, only 50 differentially methylated regions were identified. Almost all of these loci represent enhancers driving gene expression in the differentiated part of the small intestine. Finally, we show that binding of the transcription factor Tcf4 correlates with hypo-methylation and demonstrate that Tcf4 is one of the factors contributing to formation of differentially methylated regions.

**Conclusions:**

Our results reveal limited DNA methylation dynamics during small intestine stem cell differentiation and an impact of transcription factor binding on shaping the DNA methylation landscape during differentiation of stem cells *in vivo*.

## Background

DNA methylation is of critical importance for proper development. Mutants in any of the enzymes responsible for this mark are lethal [1]. The mammalian DNA methylation machinery can be subdivided into two categories: DNA methylation maintenance by DNMT1 and *de-novo *DNA methylation by DNMT3a/b [2]. The combination of bisulfite treatment and high throughput sequencing (BS-Seq) made it possible to assess the dynamics of DNA methylation during differentiation and other processes on the single nucleotide level. Initial single nucleotide resolution genome wide studies both *in vitro *and *in vivo *established the inverse relationship between methylation of histone 3 lysine 4 and DNA methylation at the transcriptional start site (TSS), but also at intergenic regions [3,4]. Furthermore, transcription factor (TF) binding sites were found to be often hypo-methylated [4]. These studies gave the first hints to what shapes the DNA methylation landscape during differentiation.

DNA methylation dynamics at TSSs during *in-vitro *differentiation of both embryonic stem cell and progenitor cells to differentiated cells has previously been investigated using MeDIP combined with microarray hybridization. In these studies, depending on the differentiation step, somewhere between 66 and >1,000 TSSs displayed differential DNA methylation levels [5,6]. As expected, the gain in DNA methylation often negatively correlated with gene expression levels [5,6]. The first genome-wide BS-seq studies focusing on differentiation of stem cells addressed ES cell (ESC) differentiation *in vitro *[3,4]. These studies revealed that upon differentiation large hypo-methylated regions are formed and many TSSs change their methylation status, reflecting their activation or inactivation during the differentiation process. Later studies focused on the differentiation of hematopoietic stem cells. These studies identified numerous differentially methylated regions (DMRs) upon differentiation, many of them associated with transcriptional start sites (TSS) [4,7,8]. In the hematopoietic system a subset of DMRs located at TSSs were in fact widening of already existing hypo-methylated regions [7]. Furthermore, hematopoietic differentiation is impaired in Dnmt3a mutants [9], suggesting a role for *de-novo *DNA methylation during differentiation in this system. These studies have created a general picture in which stem cell differentiation is accompanied by substantial DNA methylation changes. It should however be kept in mind that the number of biological systems analyzed is still small and thus generalizing statements may still be premature. In fact, recent work by the Meissner lab, using reduced representation bisulfite sequencing, has shown that during adult stem cell differentiation DNA methylation dynamics is more limited than expected (Bock *et al*., 2012). Still, this study reports on >2,000 significantly affected loci during skin stem cell differentiation. Finally, since it has been shown that *in-vitro *cultivation of cells can rapidly induce changes in DNA methylation patters [3], it is important to note that the Bock *et al*. and Hodges *et al*. studies are thus far the only studies addressing DNA methylation dynamics at single base resolution during differentiation in a completely *in-vivo *setting [7,10].

We therefore sought to study DNA methylation dynamics in an epithelial stem cell system that is well characterized, displays high stem cell activity, is medically relevant and can be studied completely *in vivo*. Currently, there are only very few systems that satisfy all these criteria simultaneously. We chose to study the mouse small intestinal epithelium. The mouse SI can be divided into three regions: a lower crypt compartment harboring long-lived stem cells [11,12] and the paneth cells that constitute the stem cell niche [13], a rapidly dividing transit amplifying zone and the Villus, a terminally differentiated region consisting of >90% enterocytes [14]. Lgr5 has been identified as a SI stem cell marker and transforming mutations in Lgr5+ SI cells have been shown to be highly tumorigenic [11,15]. Subsequent studies have shown that Lgr5 marks additional adult stem cell populations, for instance in the hair follicle [16,17].

A previously described Lgr5-GFP knock-in model allows the isolation of Lgr5-positive stem cells and their immediate descendants based on their GFP intensity [11]. Using this system, we established methylomes at single-base-resolution from three cell populations obtained directly, without any *in-vitro *culture steps, from the mouse SI: stem cells (GFP_High), their close descendants (GFP_Low), and terminally differentiated cells (Villus). In addition, in order to relate the methylation dynamics during differentiation to changes in DNA methylation that have been accumulated during earlier development, we compared the SI stem cell methylome with another Lgr5+ adult stem cell type from the hair bulge. Our results reveal, as expected, many loci that are differentially methylated between intestinal and hair bulge stem cells. These differences nicely reflect the differential expression patterns found in the two cell types. In contrast, we observed surprisingly stable DNA methylation patterns during SI stem cell differentiation, with no significant *de-novo *methylation of hypo-methylated sites. The only loci displaying significant DNA methylation dynamics are enhancers driving gene expression upon stem cell differentiation and we only observe tens of such loci. We also identify TCF4 as a transcription factor (TF) being both physically close to a subset of DMRs and affecting DMR formation, reflecting that TF binding is a significant factor in shaping DNA methylation patterns during SI stem cell differentiation *in vivo*.

## Results

### Methylomes from epithelial cell populations

**Figure 1:**
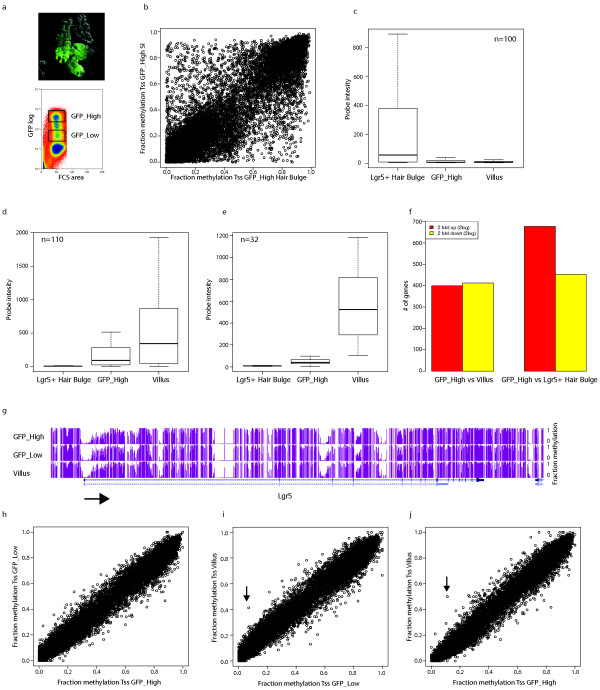
**TSS methylation dynamics**. (**a**) Fluorescent image of the Lgr5 GFP knock-in SI and a typical FACS profile. Boxes indicate GFP_High and GFP_Low populations. (**b**) All TSSs (+/- 1 kb) of which we had at least 80 CpG data-points in each methylome were analysed and their methylation status in the hair bulge (X-axis) was plotted against their methylation status in the SI (y-axis). (**c-e**) Expression of genes associated with TSSs with at least 50% DNA methylation difference between hair bulge and SI Lgr5_high population. (**c**) and (**d**) show the expression of genes with higher DNA methylation levels in the SI and hair bulge, respectively. (**e**) Displays a subset of genes from (d) that shows low to no expression in Lgr5_High, but are expressed in the Villus. In all cases only the values inside the 1.5 interquartile range are plotted. (**f**) Amount of differentially expressed genes (two-fold; 2log) between different cell types as indicated. (**g**) Genome browser view of the DNA methylation status of Lgr5 upon differentiation. Arrow indicates direction of transcription. (**h-j**) Correlation of TSS methylation between the methylomes as indicated. All TSSs (+/- 1 kb) of which we had at least 50 CpG data-points in each methylome were analysed. The one gene with a DMR on the TSS methylation is indicated with an arrow.

In order to establish methylomes of stem cells and differentiated daughter cells from the small intestinal epithelium we isolated three different populations of cells. Using FACS we isolated Lgr5-positive stem cells and their immediate descendants based on their GFP intensity [11] (Figure 1a). Fully differentiated cells were taken from the complete Villus epithelium. Importantly, all cells were subjected directly to DNA isolation without any *in-vitro *culturing steps. The purity of these cell populations can be assessed by inspection of gene expression data obtained from similarly obtained material and the potential to form organoids in culture. Indeed, we detect high expression of Lgr5 and two other known stem cell genes in the stem cell population while the expression drops rapidly upon differentiation, and we also find the expected expression differences between stem cell populations and differentiated cells for differentiation-related genes (Additional file 2, Figure S1). Finally, a previous analysis showed that only the GFP_High population has organoid-forming properties [18]. These data show that the obtained cell populations are of sufficient purity to study differential DNA methylation. We note, however, that these data do not exclude the possibility that contaminating cell populations are present in the analysed fractions and that it is possible that such contaminations limit the resolution of our DNA methylation dynamics analyses.

From the obtained cell populations DNA libraries for whole-genome bisulfite sequencing were generated. All SI methylomes are built from two independently generated, biologically distinct libraries and have an average coverage of roughly 10-fold. In addition, we derived a methylome (six-fold average coverage) from Lgr5+ stem cells that were isolated from hair bulges. Further details on library statistics can be found in the additional material (Additional file 2, Figure S2). General characteristics of mammalian methylomes also hold true for our data. For instance: gene bodies are often highly methylated while most transcriptional start sites (TSSs) are not. Furthermore CpG islands are in most cases hypo-methylated and repeat elements are hyper-methylated (data not shown).

### Differential methylation of TSSs between different Lgr5+ stem cell systems

In order to verify our approach and to set a reference for TSS-related DNA methylation dynamics, we first compared transcriptional start site (TSS) methylation of the SI stem cell with global TSS methylation of a second Lgr5+ stem cell system, the hair bulge [17] and correlated that with differences in gene expression between these two tissues. To start, we analyzed the TSS (+/- 1kb) methylation found in the two epithelial stem cell systems and represented the results in a scatter plot (Figure 1b). Clearly, many differentially methylated TSSs can be detected. In total, we found 297 TSSs with >50% DNA methylation difference between the two methylomes. In order to correlate these TSS-associated methylation changes with gene expression we derived expression data from Affimetrix expression arrays from both stem cell types. The vast majority of the genes associated with a differentially methylated TSS are differentially expressed, and lower methylation correlates strongly with higher expression (Figure 1c-e). For an overview of all significantly different expressed genes between the two different adult stem cell populations see Additional file 3, table S1. Indeed, TSS methylation is well-known to accompany the long term silencing of gene expression [19,20], suggesting that the observations we describe here reflect the long-term separation of the lineages leading to intestinal and epidermal cell fates.

Functionally, genes associated with differentially methylated TSSs, can be coupled to the stem cell system they originate from: gene ontology analysis of genes with a hypo-methylated TSS in the hair bulge reveals that these are enriched for functions in cellular response to UV and genes with a hypo-methylated TSS in the SI are enriched for annotations relating to metabolic processes (not shown).

### Lack of TSS DNA methylation dynamics during SI stem cell differentiation

We next looked at DNA methylation at TSSs during differentiation of the SI stem cell by analyzing the status of TSS methylation in the three SI-derived methylomes. We note that the number of genes that is differentially expressed between the SI stem cell and the hair bulge is very close to the number of differentially expressed genes between the SI stem cells and the Villus (Figure 1f). Thus, in terms of gene expression differences, the hair bulge and the SI stem cell are just as related to each other as are the SI stem cell and its differentiated villus cells.

First, we checked the methylation state of three well-known SI stem cell markers, Lgr5, Olfm4, and Mash2 [11,21]. Although the mRNA levels of these genes decrease >60-fold upon differentiation, their promoters do not gain methylation (Figure 1g and Additional file 2, Figure S3a, S3b). Also at a genome-wide level no significant differences were observed on the vast majority of TSSs (Figure 1h-j). To increase our sensitivity we subdivided the TSSs based on differential expression, but also this did not reveal significant differences (data not shown). To be precise, only two TSSs showed significant DNA methylation dynamics. One TSS loses DNA methylation without an accompanying change of expression of the associated gene (Additional file 2, Figure S3c and S3d). The second TSS-associated change involves the widening of an already existing hypo-methylated region (HMR). The gene associated with this TSS (Pdx1) is upregulated upon differentiation. Widening of HMRs has been described to occur frequently during HSC differentiation [7,22]. To specifically probe for HMR widening during SI differentiation we analysed the methylation dynamics of HMRs associated with TSSs. This revealed only minimal differences (data not shown).

These data show that in contrast to previously published analyses of stem cell systems [4,7,8], DNA methylation at TSSs in the SI stem cell system is very static. Interestingly, we find that the TSS of >100 genes carrying a hypo-methylated TSS in the SI stem cell, can potentially be methylated as evidenced by its methylation status in the hair bulge (Figure 1b), indicating that the lack of TSS methylation at these genes is not just reflecting the fact that many TSSs are hypo-methylated in general. Rather, these TSSs may be kept hypo-methylated for a reason. Relating to such a hypothetical reason for hypo-methylation, approximately one-third of these genes are not, or only lowly expressed in the SI stem cell, despite their hypo-methylated TSSs. Instead, these genes start to be highly expressed in the differentiated Villus cells (Figure 1e). Taken together, the lack of TSS methylation at these genes may reflect a priming of these genes to become rapidly induced during SI stem cell differentiation. Conversely, the lack of *de-novo *methylation of stem-cell specific genes upon differentiation may reflect the fast turn-over of cells in the SI epithelium, which may not allow or require the establishment of TSS hyper-methylation at 'stemness' genes upon differentiation.

### Global effects on DNA methylation during differentiation

**Figure 2:**
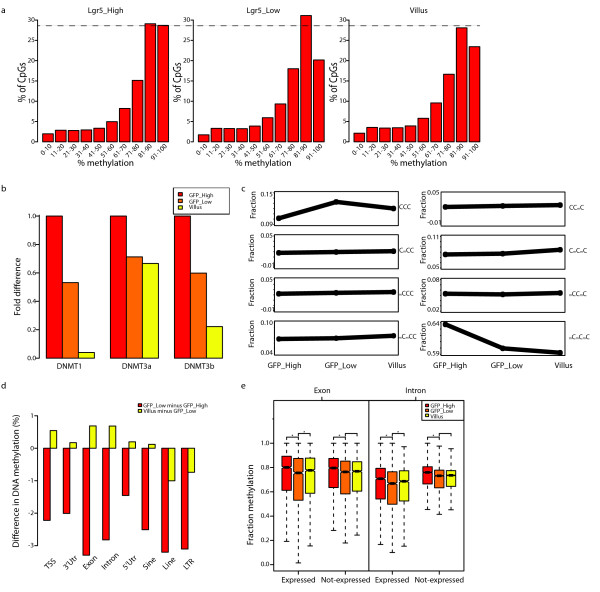
**Global DNA methylation dynamics in the SI**. (**a**) Distribution of methylation states of individual CpGs, binned according to methylation frequency (X-axis) in different methylomes as indicated. (**b**) Dnmt expression as measured on Affimetrix gene expression arrays. (**c**) Fraction of reads with indicated methylation status of the first three CpGs per read, in the different methylomes. C: unmethylated CpG; mC: methylated CpG. (**d**) DNA methylation dynamics of indicated genomic elements. Y-axis: % loss or gain of methylation average per element. (**e**) Mean methylation, per element, of expressed and non-expressed exons and introns (* *P *< 0.01, Mann-Whitney U-test).

We next analyzed DNA methylation globally. Based on studies on human ESCs [23] one might expect to find strong hypo-methylation upon differentiation of SI stem cells. However, we do not observe this; the general loss of DNA methylation is only minor (approximately 78% CpG methylation in stem cells *versus *approximately 74% in the two differentiated samples). The observed loss is confined to the fraction of highly methylated cytosines in stem cells (90-100% methylation) (Figure 2a) and coincides with a decrease in DNMT expression upon differentiation (Figure 2b) and a shortening of the cell cycle [24]. We observed that this minor loss is due to a shift from completely methylated to completely un-methylated reads (Figure 2c), indicating that neighboring CpGs lose methylation simultaneously. We then looked whether specific genomic elements (TSS, 3'UTR, intron, exon, 5'UTR, SINEs, LINEs, and LTRs) are particularly prone to loss of DNA methylation during differentiation. All these lose DNA methylation (Figure 2d), but we do detect some interesting differences. Transposable elements, as a group continuously lose DNA methylation during the whole differentiation process (Figure 2d). This does not bear functional consequences for transposon silencing, as transposon expression levels are mostly unaffected and no transposon family has specific copies that become hypo-methylated upon differentiation (Additional file 2, Figure S4a-d). In contrast, protein-coding loci either maintain or even regain methylation during terminal differentiation. Interestingly, the regain of DNA methylation at coding regions correlates with gene expression: expressed regions tend to regain methylation upon terminal differentiation (*P *<0.01, Mann-Whitney U-test) while silent regions do not (*P *>0.6, Mann-Whitney U-test) (Figure 2e). These data suggest that the relatively open chromatin structure of expressed regions may allow more efficient maintenance of methylation during proliferation and differentiation while heterochromatic loci (including transposons) do not. Everything considered, we interpret the minor loss of global DNA methylation as passive loss of DNA methylation through a combination of increased DNA synthesis and reduced DNA methylation maintenance.

### Identification of DMRs during SI stem cell differentiation

**Figure 3:**
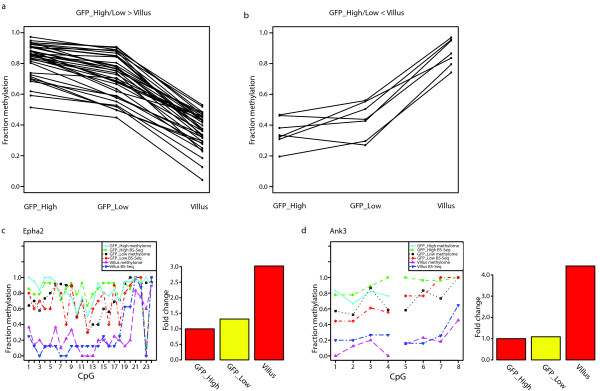
**Identification and characterization of DMRs**. (**a**, **b**) Methylation dynamics of each individual DMR called between the different methylomes. DMRs that lose (a) or gain (b) DNA methylation are displayed separately. (**c**, **d**) Confirmation of the DMRs called in the Epha2 and Ank3 genes by manual BS-Seq and their expression (Expression of GFP_high was set to one).

We continued and looked for DMRs genome-wide between the individual SI methylomes (see Methods for details). No significant DMRs could be identified between the stem cells and their first descendants. Between the stem cell and the Villus methylomes only 50 DMRs were identified, of which the majority (43) lost DNA methylation upon differentiation (Figure 3a, b). Four of these DMRs were retested using locus-specific bisulfite sequencing on independently acquired DNA samples and all four DMRs were confirmed (Figure 3c, d and Additional file 2, Figure S5a-d). We detect almost no *de-novo *DNA methylation in the three cell populations. This is consistent with the fact that Dnmt3a/b are lowly expressed in the SI system (Additional file 2, Figure S5e).

### DMRs correlate with gene expression and carry enhancer-related chromatin marks

**Figure 4:**
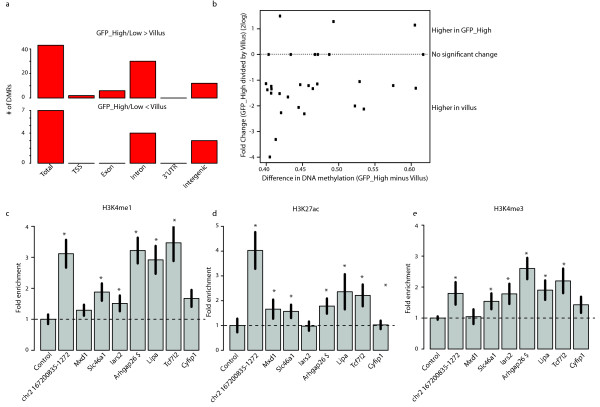
**Analysis of histone modifications and local interactions of DMRs**. (**a**) Numbers of DMRs called between methylomes and their association with different genomic regions as indicated. A DMR is labelled as TSS if within 2 kb of an annotated transcriptional start. DMRs are labelled as intergenic if they are not within 2 kb of a refseq gene. DMRs that overlap with multiple genomic elements are counted in both categories. Top graph represents DMRs that show a decrease in DNA methylation upon differentiation, lower graph depicts the DMRs with an increase during differentiation. (**b**) Scatter plot displaying the absolute difference in DNA methylation of the DMRs (GFP_High - Villus) (X-axis) *versus *the difference in expression of the associated genes between GFP_high cells and the Villus (Y-axis) (GFP_High divided by Villus). (**c-e**) Chip-qPCR results for different histone modifications found in chromatin of Villus epithelium for eight individual DMRs. DMRs are labelled after their closest gene (as indicated), or after their chromosomal location. * *P *<0.05 (student t-test). $: 4C analysis shown in, Additional file 2, Figure S6. The control is a randomly chosen region close to the Sp5 TF.

We then analyzed the association of DMRs with different genic elements (TSS/exon/intron) and the correlation of DMRs with expression of associated genes. A large fraction of DMRs (34) was found in genic elements of which, as mentioned above, only two are located at a TSS (Figure 4aand Additional file 2, Figure S3d). The few genes with DMRs in their gene body that gain methylation do not change their expression upon differentiation. In contrast, genes containing DMRs that lose methylation upon differentiation are strongly enriched for significant (*P *<0.01;student t-test) differential expression between stem cells and the Villus (21 out of 30; *P *<0.001;random permutation test) (Figure 4b). This expression change is strongly biased to upregulation upon differentiation (*P *<0.001; random permutation test).

The observation that the methylation status of non-promoter associated DMR-loci correlates inversely with gene expression made us hypothesize that these DMRs may in fact reveal gene-regulatory domains, or enhancers. Such domains can be marked by the presence of TF binding sites and by Histone H3 subunits that are mono-methylated at lysine 4 (H3K4me1), acetylated at lysine 27 (H3K27ac), and have minimal H3K4me3 [25]. Indeed, TF binding sites are indeed often mildly methylated [4,26]. To confirm the potential regulatory function of the DMRs, ChIP-qPCR was performed for H3K4me1, H3K27ac, H3K4me3, and H3K9me3 on chromatin isolated from Villus epithelium. Of the eight DMRs tested, seven show significant enrichment for H3K4me1 and six for H3K27ac (*P *<0.05; student t-test) (Figure 4c and 4d). Interestingly, six of these regions also display significant enrichment for H3K4me3 (*P *<0.05; student t-test) (Figure 4e), but drastically lower than typical enrichment values found at TSS [27,28]. Importantly, at these loci H3K9me3, a repressive mark, was not enriched (Additional file 2, Figure S6a). Furthermore, analysis of available ChIP-seq data from the total SI [29] shows enrichment of H3K4me1 and H3K27ac, but not H3K4me3 over our DMRs (Additional file 2, Figure S6b-d). These results indicate that the chromatin at the identified DMR loci is indicative of gene-regulatory functions.

### DMRs physically loop to activated genes

**Figure 5:**
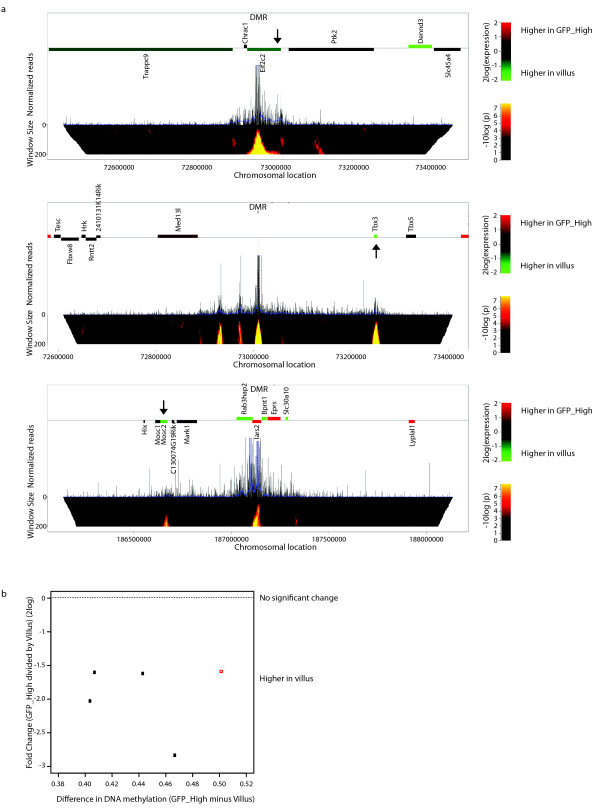
**4C-seq analysis of a subset of DMRs**. (**a**) Domainograms of interaction profiles of three DMRs looping to the TSS of a differentially expressed gene. Top panel: intragenic DMR (Eif2c2) looping to the host TSS. Middle panel: intergenic DMR looping to Tbx3. Bottom panel: intragenic DMR (Lars2) looping to a distant gene (Mosc2). Significant interactions are indicated in the domainogram using different colours, corresponding to different *P *values as indicated on the right. Above the domainogram normalized reads are plotted. Genes are shown as solid horizontal bars. The vertical arrows indicate the significant 4C interaction with a TSS. Significant differentially expressed genes *P *<0.01 (student t-test) are color-coded as indicated on the right. (**b**) Scatter plot displaying the absolute difference in DNA methylation of the DMRs (GFP_High - Villus) (X-axis) *versus *the difference in expression of the gene, which it physically associates with based on 4C analysis, between GFP_High cells and the Villus (Y-axis) (GFP_High divided by Villus). Black circles indicate gene expression changes with *P *<0.01 (student t-test) and the red square *P *<0.05 (student t-test).

To further test the hypothesis that the identified DMRs represent enhancers we used 4C-seq analyses to look into the association of the DMRs with TSSs of differentially expressed genes [30]. Importantly, this technique will identify associations between a specified locus (the DMR) with any other genomic locus, allowing us to ask the open question: can we detect specific loci that are frequently in close contact with one specified DMR? We first applied this technique to 11 intragenic DMRs. Four of these can be shown to contact the TSS of the gene containing the DMR (Figure 5a (upper panel), Additional file 2, Figure S7). Interestingly, one of the intragenic DMRs that does not contact the TSS of the gene in which it resides in fact makes highly significant contacts with the TSS of another gene located approximately 450 kb away (Figure 5a (lower panel)). Consistent with the idea that DMRs are enhancers driving expression in the Villus, this distal gene, Mosc2, and not the gene carrying the DMR, is three-fold upregulated upon differentiation. Next, we analyzed intergenic DMR loci. A significant number of these, five out of nine tested, reveal contacts with the TSS of a neighboring gene. In all five cases the contacted gene is up-regulated in the Villus compartment (Figure 5a (middle panel), Figure 5b, Additional file 2, Figure S7). Taken together, our data strongly argue that the regions displaying DNA methylation dynamics in differentiating SI stem cells are in fact enhancers that drive gene expression upon differentiation.

### Tcf4 frequently binds close to DMRs

**Figure 6:**
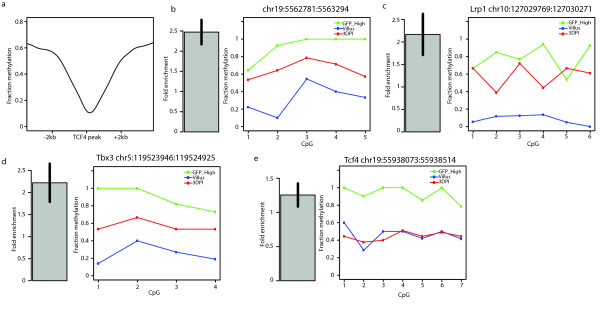
**Tcf4 induces DMR formation in the Villus**. (**a**) Global depletion of DNA methylation at Tcf4 binding sites at least 5 kb away from a TSS. (**b-e**) Left panel indicates tested CpGs of different DMRs after Tcf4 ablation in the SI and the values found in the methylomes. Above the panels the genomic coordinates of the tested DMR is indicated and if applicable the gene it is located in or contacts by looping. Right panel shows ChIP-qPCR enrichments of TCF4 at the DMR tested in the panel to the left. 3DPI: 3 days post induction.

To further look into a potential cause driving the formation of DMRs we asked whether TF binding sites are found close to DMRs. For this question we focussed on Tcf4, a critical TF in the epithelium of the SI [31,32]. A ChIP-seq dataset derived from isolated crypts (the stem cells plus their niche) was already available for this TF (not shown). We first checked the DNA methylation status of non-TSS associated Tcf4 peaks in the SI stem cell methylome and found that Tcf4 binding sites are on average hypo-methylated, (Figure 6a). Virtually identical patterns are observed in the other two SI methylomes (not shown). These data suggest a role for Tcf4 or Tcf4 interacting proteins in shaping the methylation status of the DNA it binds to, an observation in line with previous publications [4,26].

We then checked the proximity of Tcf4 binding sites to DMRs. We could identify multiple DMRs within 1 kb of a Tcf4 binding sites. Six of these were in gene bodies (Iars2, Lrp1, Slc46a1, Ago2, Myo5b, and Tcf4 itself). We then tested whether Tcf4 binds to these loci also in the Villus using ChIP-qPCR. For six out of seven tested loci we could confirm the interaction (Figure 6b-e and Additional file 2, Figure S8a-c), indicating that Tcf4 can often be found binding close to a locus that loses DNA methylation during differentiation.

### Tcf4 contributes to DMR formation

To probe the effect of Tcf4 in the formation of DMRs we asked whether loss of Tcf4 would affect the methylation status at the identified DMRs. A complication to this question is that a complete knock-out of Tcf4 in the SI results in severe proliferation defects, preventing a meaningful analysis [32]. We therefore made use of a conditional Tcf4 knock-out model in which p450 driven Cre-recombinase induces Tcf4 disruption upon b-naphtoflavone injection. This system is not 100% efficient [33]. Three days after induction of CRE the majority of the cells, but not all, have recombined and the Villus epithelium looks grossly normal (Additional file 2, Figure S8d-h), allowing meaningful comparison of wild-type and Tcf4 mutant tissue.

At this time point after induction of Tcf4 deletion, the DNA methylation status of six Tcf4 associated DMRs and one non-Tcf4-associated DMR was analyzed by manual BS-sequencing (Figure 6b-e and Additional file 2, Figure S8a-c). This analysis showed that five of these DMRs are more strongly methylated in the Villus upon loss of Tcf4. Two loci did not show an increase in DNA methylation upon loss of Tcf4, one of them being the locus not bound by Tcf4 in the villus (Tcf4 itself; Figure 6e). Although direct effects of DNA methylation on enhancer function cannot be extracted from these data, they are in line with the idea that Tcf4, or Tcf4 binding partners at enhancers can induce hypo-methylation upon differentiation, potentially by binding to these sites and interfering with the DNA methylation machinery. We note that expression of Tcf4 does not change significantly during SI stem cell differentiation (Additional file 2, Figure S8i) and that Tcf4 already binds to these loci in the stem cell compartment, as indicated by ChIP-seq signals for Tcf4 at these loci, suggesting that differential binding of Tcf4 itself during differentiation may not directly drive DMR formation. Rather, the differential recruitment of factors through Tcf4 may be a more plausible explanation for the observed results. We note, however, that our results do not necessarily reflect direct effects of DNA binding of Tcf4 and/or its co-factors.

## Discussion

We describe single-base-resolution methylation analysis of an epithelial adult stem cell and its descendants. In contrast to studies describing the hematopoietic and ESC systems we do not observe widespread TSS methylation dynamics upon differentiation. TSS methylation in the stem cell already reflects the methylation status found in its differentiated descendants, suggesting that the epi-genome in the stem cell may be to some extent primed for differentiation into SI epithelial cells. Also, genes that become silent upon differentiation do not attract methylation on their TSSs, suggesting that the SI epithelium does not require a locking-in of gene expression status through DNA methylation. The few effects on DNA methylation that we do see are restricted to a set of enhancers that drive gene expression upon differentiation. At these loci, loss of DNA methylation appears to be promoted through TF binding. Below we will discuss our findings in more detail.

### Absence of TSS methylation dynamics during differentiation

We detect little to no dynamics concerning the methylation of TSSs within the SI epithelium. This differs from what has been observed in other *in-vitro *and *in-vivo *differentiation studies [4-8,22,26]. To our knowledge, these studies consistently report significantly higher numbers of DMRs than we detect in the SI. The fact that we do not observe *de-novo *DNA methylation with a direct link to differentiation raises the question whether there is a role for Dnmt3a/b in the small intestine. In this light, the observation that Dnmt3b knock-out intestine is phenotypically indistinguishable from wild-type is interesting [34]. In fact, *de-novo *methylation might be harmful in this system, as overexpression of DNMT3b is associated with increased formation of colonic adenomas [35], while Dnmt3b deletion prevents neoplasia formation [34]. We do not know the reasons behind these deleterious effects of DNMT3b but one possibility might be that it may block the activation of genes to should be activated during differentiation. More detailed methylome analysis of methylomes during early stages of tumorigenesis will be required to address this question. On the other hand, some genes associated with SI differentiation already display hypo-methylation of their TSS in the SI stem cells. Given that their TSSs can be hyper-methylated in the hair bulge, this finding indicates that there is a group of genes that is already pre-specified in the SI stem cell to be turned on during differentiation.

In a recent study, Bock *et al*. (2012) have analyzed the DNA methylation status of several adult stem cell populations, including those of the skin. These authors also find modest DNA methylation dynamics, although they do detect significantly more DMRs during skin differentiation than we report here. The reasons behind these differences remain to be explored, but perhaps the longer lifetime of differentiated skin cells requires more extensive DNA methylation dynamics in order to maintain proper gene expression profiles.

### Only few enhancers trigger DMR formation

We demonstrate that the few DMRs that arise during differentiation of SI stem cells define enhancers that drive gene expression during differentiation. Still, the question remains why only so few enhancers trigger DMR formation. Part of the answer might be related to the lack of TSS methylation dynamics. If indeed the SI stem cell genome is primed for expression of differentiation-related genes, one would expect this not to be restricted to TSSs, but to also extend to gene-regulatory domains. This would imply that many enhancers are already bound by the required TFs and/or other associated proteins, leading to hypo-methylation already in the stem cells, and thus a lack of DMRs formation upon differentiation. In fact, such a scenario was recently shown for Foxp3 and the glucocorticoid receptor in other systems [36,37].

### Impact of Tcf4 on DNA methylation

We find that Tcf4 binding sites are generally hypo-methylated in SI epithelial cells. This is consistent with previous reports describing hypo-methylation induced by TF binding in *in-vitro *conditions [4,26,38]. Our results demonstrate that similar effects can be identified *in vivo*, although in all these studies, including ours, the actual direct effects of DNA binding has not been addressed. In addition, we find that Tcf4 has an impact on DMR formation during differentiation. This is intriguing, since Tcf4 itself appears to be present and bound close to the DMRs both before and during differentiation, suggesting that the effect of Tcf4 on DMR formation is indirect. In that respect it is interesting to note that Tcf4 binding sites are most often not situated directly within DMRs, but rather flank DMRs (not shown). This may indicate that in the process of differentiation, Tcf4 recruits additional factors that may bind to sites flanking Tcf4 and that these additional factors affect DNA methylation. The identification of complexes recruited by Tcf4 to these sites during differentiation of SI stem cells will be required to test this hypothesis.

### DMR-associated genes

As discussed above, only a limited set of enhancers loses DNA methylation during differentiation. Is there anything unusual about the genes regulated by these enhancers? One gene regulated by such an enhancer, Tcf4, has significant impact on the system, as knock-out models display SI-related phenotypes [31,32,39]. In fact, as discussed above, Tcf4 is itself a driver behind DMR formation. Two other noteworthy genes associated with a DMR-enhancer are Mxd1, a negative regulator of the Wnt responsive gene c-Myc, and Eif2c2/Ago2, one of the key players in the miRNA pathway [40,41]. SI phenotypes associated with these genes have not yet been described, but both genes are well-known proteins related to cell proliferation and differentiation. Finally, we identified Tbx3 as a gene that is contacted by such an enhancer. Tbx3 is a TF and mutations in Tbx3 have been identified as responsible for the development of ulnar-mammary syndrome in humans [42]. Although not a disease affecting the SI, it does illuminate the strong impact that Tbx3 can have on homeostasis and development.

Thus, many of the genes regulated by these DMR-enhancers have strong effects on development and differentiation. It may be that the enhancers that can drive the expression of genes with a particularly strong impact on differentiation are under very strict control and that the system does not tolerate pre-occupation of such enhancers in pre-differentiation stages. It will thus be interesting to determine the impact of these enhancers and their associated genes on the homeostasis of the small intestine.

## Conclusions

Our data show that during the differentiation of the SI stem cell only a minimal amount of DMRs arise. Furthermore our results suggest a role for TCF4 in the formation of a subset of DMRs.

## Material and methods

### ChIP-qPCR

Villus epithelium was isolated by incubation of small intestine that was cut into small pieces in PBS supplemented with 1mM EDTA/EGTA. The small intestine was transferred to fresh buffer every 10 min, leaving behind detached epithelium. This procedure was repeated up to 14 times. In general, the first fractions contain Villus and the last fractions contain Crypts. Purity was checked by conventional microscopy. ChIP was performed with antibodies against H3K4me1 (abcam), H3K4me3 (abcam), H3K9me3 (abcam), H3K27ac (abcam), and Tcf4 (Santa Cruz) as described previously [43], but with the following modifications. Villus was fixed in 1% formaldehyde for 30 min at RT. Washing was performed as described, but to prepare the chromatin the cell lysis step was skipped and nuclear lysis was performed directly. From these lysates chromatin was isolated by phenol/chloroform extraction and quantified by gel electrophoresis and NanoDrop (NanoDrop Technologies, Wilmington, DE, USA). Chromatin was sheared using a covaris S2 apparatus. ChIPs with the different antibodies were performed in parallel with 4-6 ug of antibody on 5-10 ug chromatin. After incubation at 4C for 4-5 h beads were washed five times in RIPA buffer.

Quantitative PCR was conducted on a Biorad ICycler system with SYBR Green. Normalized enrichment values were calculated with a standard formula. For primer sequences see Additional file 1, Table S2.

### 4C

Template generation for 4C analysis on Villus nuclei was performed essentially as described [44]. For this study we used different combination of primary and secondary cutters for different viewpoints. To increase short distance resolution two four cutters were used in all experiments. Colom purified PCR products were submitted for sequencing on the Illumina HISeq 2000 genome analyzer.

### Library preparations

FACS analysis was performed as described previously [18]. In short: to enrich for crypt epithelium small intestine from *Lgr5-EGFP-IRES-CreERT2 *mice was incubated in PBS supplemented with 1mM EDTA/EGTA as described above. Crypts were subsequently incubated in Trypsin (10 mg/mL) and DNAse (0.8 µg/µL) for 30 min at 37ºC. Single cells were obtained by filtering through a 40 µm mesh and GFP expressing cells were isolated using a MoFlo cell sorter (DAKO). DNA from 350 K (GFP_High) or 150 K (GFP_Low) cells was isolated as follows: cells were lysed in lysis buffer (10 mM Tris (pH8), 100 mM EDTA, 1% SDS, and 1 ug/mL protK) followed by standard phenol/chloroform extraction and ethanol precipitation. Villus epithelium was isolated as described above and also subjected to the treatment describe above to isolate DNA. Lgr5 positive stem cells from the skin were isolated similarly as done for SI except that the incubation with Trypsin (10 mg/mL) was extended in some cases up to 4 h. The hair bulge was pulled out of the skin by scrapping. DNA was then sonicated to approximately 150-400 base pairs using the Covaris S2 sonicator. The sheared DNA was separated into three portions and adapter ligation was performed as described [7]. Separate ligation products were bisulfite treated (see below) and amplified by 16 PCR cycles. Thereafter PCR products were pooled again and submitted for sequencing on an Illumina GA2X genome analyzer.

### Manual BS-Seq

DNA was isolated as described under library preparations. Between 100-500 ng of genomic DNA was subjected to bisulfite conversion using the EZ-DNA Methylation_Gold Kit (Zymo research). Primers were designed using MethPrimer [45]. For a list of primers used in this study see Additional file 1, Table S2. PCR Amplified fragments were sub cloned using the TA-TOPO kit (Invitrogen) and transformed. Individual clones were subsequently sequenced. In all cases described at least 10 individual clones were assayed.

### Expression arrays of Lgr5+ cells obtained from the SI and the hair bulge

Microarray analysis was essentially done as described [46] in short: RNA from Lgr5_High SI (four), Lgr5_low (three), Villus (three), and Lgr5_Low Hair bulge (three) was isolated after FACS as described above (number between brackets indicate amount of biological replicates performed). Approximately 300,000 to 500,000 cells were sorted for each microarray experiment. RNA concentration and quality was determined using a NanoDrop (NanoDrop Technologies, Wilmington, DE, USA) and Agilent 2100 Bioanalyzer (Agilent Technologies, Palo Alto, CA, USA), respectively. Fragmentation of cRNA, hybridization to genome-wide mRNA expression platform harboring 20,819 unique genes (Affymetrix HT MG-430 PM Array Plate) and scanning was carried out according to the manufacturer's protocol (Affymetrix Inc., Santa Barbara, CA, USA) at the MicroArray Department of the AMC. With the RMA-sketch algorithm from Affymetrix Power Tools intensity values and confidence intervals were assigned to probe-sets.

### Computational analysis

DMR and HMR calling was done as described previously [7,22]. Additionally, DMRs were filtered asking for at least 10 CpGs and 40% methylation difference.

### 4C analysis

The mapping and data analysis was carried out similar to Splinter *et al. *[30,47].

However, a high-resolution 4C experiment generates substantial number of fragment ends that are formed between two restriction sites of the first restriction enzyme only, so-called 'blind' fragments. The blind fragments have a different frequency distribution compared to regular fragments. We therefore performed quantile normalization using the Limma package in R to make the distribution of both sets of fragment ends identical. To identify regions with significant enrichment of 4C signal we generate local 4C domainograms on these normalized datasets. In these graphs genomic windows of a given are compared to their directly flanking genomic windows using a Wilcoxon rank-sum test. Formally, the window *W_i..i+w-1 _*is compared to the windows *W_i-w..i-1 _*and *W_i+w..i+2w-1_*, where *W *is a genomic window, *i *is the index of a fragment end in the genome and *w *is the size of the window. A sliding window approach is employed to calculate the enrichment along the site of the 4C analysis. By calculating the statistical test over a range of window sizes and plotting the resulting -log_10 _transformed matrix of *P *values along the chromosome a multiscale representation of the 4C data is obtained.

Furthermore to confirm the self-regulatory mechanism of DMRs directly associated with genes that are upregulated upon differentiation only looping from the viewpoint to the TSS of the same gene was assayed. For short range interaction manual inspection of peaks was performed. In the case where orphan DMRs were linked to genes all interaction 750 kb up- and downstream of the viewpoint were essayed. Looping interacting within 5 kb from a TSS were filtered out and linked to expression data.

### Animal experiments

All experiments with animals were conducted according to the local regulations and with permission of the local animal welfare officers.

### Data Access

The BS-seq and the microarray data from this study have been submitted to the NCBI Gene Expression Omnibus (GEO) (http://www.ncbi.nlm.nih.gov/geo/) under accession nos. SRP020633 and GSE46303, respectively.

## Abbreviations

4C-seq: Circularized Chromosome Conformation Capture-sequencing; BS-Seq: Bisufite sequencing; ChIP-qPCR: Chromatin Immunoprecipitation-quantitative real-time polymerase chain reaction; ChIP-seq: Chromatin Immunoprecipitation-sequencing; DMRs: differentially methylated region; ESC: embryonic stem cell; FACS: Fluorescence-activated cell sorting; GFP: green fluorescent protein; H3: histone 3; HMR: hypo-methylated region; HSC: hematopoietic stem cell; MeDIP: methylated DNA immunoprecipitation; SI: small intestine; TSS: transcriptional start site; TF: transcription factor; UV: ultraviolet.

## Competing interests

The authors declare that they have no competing interests.

## Authors' contributions

LJTK, MvdW, ADS, and RFK designed the study. Bioinformatic analysis was performed by FF, BD, and ADS. 4C bioinformatic analysis by HVDW, EDW, and WDL. FACS sorting was performed by MvdW. 4C, Library preparation, ChIP-qPCR, and Manual BS-Seq was done by LJTK. Illumina sequencing was performed by AM and GJH. The manuscript was written by LJTK and RFK with input from HC, AM, ADS, GJH, HVDW, EDW, and MvdW. All authors read and approved the final manuscript.

## Supplementary Material

Additional file 1 A more detailed description of material and methods accompanying the manuscript. **Table S2**. List of primer sequences used in this study. **Table S3**. List of the 4C primer sequences used in this study. **Legends to Figures S1-S8** Click here for file

Additional file 2 **Figure S1: Expression of marker genes in the purified cell populations**. Expression of a set of marker genes, derived from Affimetrix micro-array experiments, in the three sorted cell populations. **Figure S2: Mapping statistics**. Statistics on the high-throughput sequencing described in this manuscript. **Figure S3: TSS methylation and gene expression in the SI**. Figure showing genome browser view of different genes. **Figure S4: No changes in HMR distribution or expression of transposons**. Figure showing the lack of HMR distribution at transposons. **Figure S5: Two examples of confirmation of DMRs called between the methylomes**. Manual BS-Seq conformation of two DMRs called in the methylomes. **Figure S6: Additional ChIP-qPCR and ChIP-Seq analysis**. Chip-qPCR and Chip-seq analysis of the DMRs called between SI stem cells and villus. **Figure S7: 4C domainograms of three intergenic DMRs**. Additional 4C experiments performed on DMRs called between SI stem cells and villus. **Figure S8: A role for TCF4 in DMR formation**. Additional data implication TCF4 in DMR formation in the SI. Click here for file

Additional file 3 **Table S1**. List of genes differentially expressed between the SI and hair bulge stem cell compartment. Click here for file
