# Supplementary material for: DNA methylation dynamics during intestinal stem cell differentiation reveals enhancers driving gene expression in the villus
Source: Genome Biol. 2013 May 28;14(5):R50. doi: 10.1186/gb-2013-14-5-r50 (PMC4053812; doi:10.1186/gb-2013-14-5-r50)
Supplement: Additional file 1 — A more detailed description of material and methods accompanying the manuscript. Table S2. List of primer sequences used in this study. Table S3. List of the 4C primer sequences used in this study. Legends to Figures S1-S8 [file gb-2013-14-5-r50-S1.DOCX]

Additional data to:

**DNA methylation dynamics during intestinal stem cell differentiation reveals enhancers driving gene expression in the villus.**

Lucas J T Kaaij^1^, Marc van de Wetering^1^, Fang Fang^3^, Benjamin Decato^3^, Antoine Molaro^2^, Harmen J G van de Werken^1^, Johan H van Es^1^, Jurian Schuijers^1^, Elzo de Wit^1^, Wouter de Laat^1^, Gregory J Hannon^2^, Hans C Clevers^1^, Andrew D Smith^3^ and René F Ketting^1,4,^*

^1^ Hubrecht Institute-KNAW & University Medical Centre Utrecht, Uppsalalaan 8, 3584 CT Utrecht, The Netherlands

^2^ Howard Hughes Medical Institute, Cold Spring Harbor Laboratory, 1 Bungtown Road, Cold Spring Harbor, NY 11724, USA

^3^ Molecular and Computational Biology, University of Southern California, Los Angeles, CA 90089, USA

^4^ Institute for Molecular Biology, Ackermannweg 4, D-55128 Mainz, Germany

- To whom correspondence should be addressed. E-mail: r.ketting@imb-mainz.de

Keywords: DNA methylation, Adult stem cell differentiation.

**Methods**

**ChIP-qPCR**

Villus epithelium was isolated by incubation of small intestine that was cut into small pieces in PBS supplemented with 1mM EDTA/EGTA. The small intestine was transferred to fresh buffer every 10 minutes, leaving behind detached epithelium. This procedure was repeated up to 14 times. In general, the first fractions contain Villus and the last fractions contain Crypts. Purity was checked by conventional microscopy. ChIP was performed with antibodies against H3K4me1 (abcam), H3K4me3 (abcam), H3K9me3 (abcam), H3K27ac (abcam) and TCF4 (Santa Cruz) as described previously [[1](#_ENREF_1)], but with the following modifications. Villus was fixed in 1% formaldehyde for 30 min at RT. Washing was performed as described, but to prepare the chromatin the cell lysis step was skipped and nuclear lysis was performed directly. From these lysates chromatin was isolated by phenol/chloroform extraction and quantified by gel electrophoresis and NanoDrop (NanoDrop Technologies, Wilmington, DE, USA). Chromatin was sheared using a Covaris S2 apparatus. ChIPs with the different antibodies were performed in parallel with 4-6 ug of antibody on 5-10 ug chromatin. After incubation at 4C for 4-5 hours beads were washed 5 times in RIPA buffer.

Quantitative PCR was conducted on a Biorad ICycler system with SYBR Green. Normalized enrichment values were calculated with a standard formula. For primer sequences see Table S2.

**4C**

Template generation for 4C analysis on Villus nuclei was performed essentially as described [[2](#_ENREF_2)]. For this study we used DpnII and Csp6I as the primary and secondary cutter, respectively. To increase short distance resolution two four cutters were used in all experiments. Colom purified PCR products were submitted for sequencing on the Illumina HISeq 2000 genome analyser [[3](#_ENREF_3)]. See separate table for primer sequences used in this study (see below Table S3).

**Library preparations**

FACS analysis was performed as described previously [[4](#_ENREF_4)]. In short: to enrich for crypt epithelium small intestine from *Lgr5-EGFP-IRES-CreERT2* mice was incubated in PBS supplemented with 1mM EDTA/EGTA as described above. Crypts were subsequently incubated in Trypsin (10 mg/ml) and DNAse (0.8 µg/µl) for 30 minutes at 37ºC. Single cells were obtained by filtering through a 40µm mesh and GFP expressing cells were isolated using a MoFlo cell sorter (DAKO). DNA from 350K (GFP_High) or 150K (GFP_Low) cells was isolated as follows: cells were lysed in lysis buffer (10mM Tris (pH8), 100mM EDTA, 1% SDS, and 1ug/ml protK) followed by standard phenol/chloroform extraction and ethanol precipitation. Villus epithelium was isolated as described above and also subjected to the treatment describe above to isolate DNA. DNA was then sonicated to approximately 150-400 base pairs using the Covaris S2 sonicator. The sheared DNA was separated into three portions and adapter ligation was performed as described [[5](#_ENREF_5)]. Separate ligation products were bisulfite treated (see below) and amplified using 16 cycles. Thereafter PCR products were pooled again and submitted for sequencing on an Illumina GA2X genome analyzer.

**Generation of compound mice**.

The transgenic line p450^Cre^ was crossed with Tcf4^LoxP^ mice to obtain strain p450^Cre^_Tcf4^LoxP/LoxP^ mice. The Cre enzyme was induced by a single intra-peritoneal injection on day 0 of 200 μl b-napthoflavone (5 mg/200 μl; Sigma-Aldrich) dissolved in sunflower oil. The 6- to 12-week-old mice were sacrificed, and the intestines were isolated 3 days after cre induction. All procedures were performed in compliance with local animal welfare laws, guidelines, and policies.

**Manual BS-Seq**

DNA was isolated as described under library preparations. Between 100-500 ng of genomic DNA was subjected to bisulfite conversion using the EZ-DNA Methylation_Gold Kit (Zymo research). Primers were designed using MethPrimer [[6](#_ENREF_6)]. For a list of primers used in this study see Table S2. PCR Amplified fragments were sub cloned using the TA-TOPO kit (Invitrogen) and transformed. Individual clones were subsequently sequenced. In all cases described at least 10 individual clones were essayed.

In the case where the role of TCF4 in DMR formation was essayed. The manual BS-Seq was performed on two SIs and the methylation data was afterwards combined.

**Expression arrays of Lgr5+ cells obtained from the SI and the hair-buldge**

# Microarray analysis was essentially done as described [[7](#_ENREF_7)] in short: RNA from Lgr5_High SI (four), Lgr5_low (three), Villus (three) and Lgr5_Low Hair buldge (three) was isolated after FACS as described above (number between brackets indicate amount of biological replicates performed). Approximately 300,000-500,000 cells were sorted for each microarray experiment. RNA concentration and quality was determined using a NanoDrop (NanoDrop Technologies, Wilmington, DE, USA) and Agilent 2100 Bioanalyzer (Agilent Technologies, Palo Alto, CA, USA), respectively. Fragmentation of cRNA, hybridization to genome-wide mRNA expression platform harboring 20,819 unique genes (Affymetrix HT MG-430 PM Array Plate) and scanning was carried out according to the manufacturer's protocol (Affymetrix Inc., Santa Barbara, CA, USA) at the MicroArray Department of the AMC. With the RMA-sketch algorithm from Affymetrix Power Tools intensity values and confidence intervals were assigned to probe-sets. The data was visualized and analyzed using the R2 web application, which is available at ([http://r2.amc.nl](https://mail.hubrecht.eu/owa/redir.aspx?C=4af28b89a34c44e69fe2b88c52fea022&URL=http%3a%2f%2fr2.amc.nl%2f)).

**Computational analysis**

DMR and HMR calling was done as described previously [[5](#_ENREF_5), [8](#_ENREF_8)]. Additionally, DMRs were filtered asking for at least 10 CpGs and 40% methylation difference.

**4c analysis**

The mapping and data analysis was carried out similar to Splinter et al[[9](#_ENREF_9)].

However, a high-resolution 4C experiment generates substantial number of fragment ends that are formed between two restriction sites of the first restriction enzyme only, so-called “blind” fragments. The blind fragments have a different frequency distribution compared to regular fragments [[3](#_ENREF_3)]. We therefore performed quantile normalization using the Limma package in R to make the distribution of both sets of fragment ends identical. To identify regions with significant enrichment of 4C signal we generate local 4C domainograms on these normalized datasets. In these graphs genomic windows of a given are compared to their directly flanking genomic windows using a Wilcoxon rank-sum test. Formally, the window *W_i..i+w-1_* is compared to the windows *W_i-w..i-1_* and *W_i+w..i+2w-1_*, where *W* is a genomic window, *i* is the index of a fragment end in the genome and *w* is the size of the window. A sliding window approach is employed to calculate the enrichment along the site of the 4C analysis. By calculating the statistical test over a range of window sizes and plotting the resulting -log_10_ transformed matrix of p-values along the chromosome a multiscale representation of the 4C data is obtained.

Furthermore to confirm the self-regulatory mechanism of DMRs directly associated with genes that are upregulated upon differentiation only looping from the viewpoint to the TSS of the same gene was essayed. For short range interaction manual inspection of peaks was performed. In the case where orphan DMRs were linked to genes all interaction 750 kb up and downstream of the viewpoint were essayed. Looping interacting within 5 kb from a TSS were filtered out and linked to expression data.

**Animal experiments**

All experiments with animals were conducted according to the local regulations and with permission of the local animal welfare officers.

**References**

1. Wardle FC, Odom DT, Bell GW, Yuan B, Danford TW, Wiellette EL, Herbolsheimer E, Sive HL, Young RA, Smith JC: **Zebrafish promoter microarrays identify actively transcribed embryonic genes.** *Genome biology* 2006, **7:**R71.

2. Simonis M, Kooren J, de Laat W: **An evaluation of 3C-based methods to capture DNA interactions.** *Nature methods* 2007, **4:**895-901.

3. van de Werken HJ, Landan G, Holwerda SJ, Hoichman M, Klous P, Chachik R, Splinter E, Valdes-Quezada C, Oz Y, Bouwman BA, et al: **Robust 4C-seq data analysis to screen for regulatory DNA interactions.** *Nature methods* 2012, **9:**969-972.

4. Sato T, Vries RG, Snippert HJ, van de Wetering M, Barker N, Stange DE, van Es JH, Abo A, Kujala P, Peters PJ, Clevers H: **Single Lgr5 stem cells build crypt-villus structures in vitro without a mesenchymal niche.** *Nature* 2009, **459:**262-265.

5. Hodges E, Molaro A, Dos Santos CO, Thekkat P, Song Q, Uren PJ, Park J, Butler J, Rafii S, McCombie WR, et al: **Directional DNA methylation changes and complex intermediate states accompany lineage specificity in the adult hematopoietic compartment.** *Molecular cell* 2011, **44:**17-28.

6. Li LC, Dahiya R: **MethPrimer: designing primers for methylation PCRs.** *Bioinformatics* 2002, **18:**1427-1431.

7. Revet I, Huizenga G, Chan A, Koster J, Volckmann R, van Sluis P, Ora I, Versteeg R, Geerts D: **The MSX1 homeobox transcription factor is a downstream target of PHOX2B and activates the Delta-Notch pathway in neuroblastoma.** *Experimental cell research* 2008, **314:**707-719.

8. Molaro A, Hodges E, Fang F, Song Q, McCombie WR, Hannon GJ, Smith AD: **Sperm methylation profiles reveal features of epigenetic inheritance and evolution in primates.** *Cell* 2011, **146:**1029-1041.

9. Splinter E, de Wit E, Nora EP, Klous P, van de Werken HJ, Zhu Y, Kaaij LJ, van Ijcken W, Gribnau J, Heard E, de Laat W: **The inactive X chromosome adopts a unique three-dimensional conformation that is dependent on Xist RNA.** *Genes & development* 2011, **25:**1371-1383.

**Additional tables**

**Table S1 List with genes being differentially expressed between SI LGR5_High and LGR5_Low Hair bulge.**

See separate excel sheet.

**Table S2 List with primer sequences used**

| **Tet qPCR** |  |
| --- | --- |
| Tet1_F | GAGCCTGTTCCTCGATGTGG |
| Tet1_R | CAAACCCACCTGAGGCTGTT |
| Tet2_F | TGTTGTTGTCAGGGTGAGAATC |
| Tet2_R | TCTTGCTTCTGGCAAACTTACA |
| Tet3_F | CCGGATTGAGAAGGTCATCTAC |
| Tet3_R | AAGATAACAATCACGGCGTTCT |
| B2m_F | CTTCAGTCGTCAGCATGG |
| B2m_R | GTTCTTCAGCATTTGGATTTC |
|  |  |
| **Manual bisulfite PCR** |  |
| Ank3_F _1 | TTTTGGATTTTTGTTTTAGGAAATG |
| Ank3_R _1 | AAATACTAACCTCTCTCATACCCCC |
| Ank3_F _2 | GAGATATAAGGTGATTAGTTTGGGTTATTA |
| Ank3_R _2 | AAACCATTTCCTAAAACAAAAATCC |
| Epha2_F | TGGGATTTGATGTAGAATATTATGGA |
| Epha2_R | TAAAAAACCAAATAAAAACCCTTCC |
| Cyfip1_F | GTTAAAATAGTGTAGTTGTAATTGAGGAAA |
| Cyfip1_R | ATCAATAAATAATAAACCTCAAAACCTAAA |
| Pdx-1F_1 | GGGTGAATAGGGAGAGGTTGTATTAA |
| Pdx-1R_1 | TCAAAAAAAATTAACACCCCAAAAA |
| Pdx-1F_2 | TTGGATTGTAAATAGTTGTTTTTTTT |
| Pdx-1R_2 | CACCCTTAAATCCTTAATAAACCTC |
| Ifi202_F | TTTAGATTTTTAATAAGGTAGAGTGTGTTA |
| Ifi202_R | ACCTATCATAATTTCTCAAAATAACTACTA |
| Krt16_F | TTTTTTAGTTAGGATGGAGGATATG |
| Krt16_R | ACTAAAATCAACAATTAAAAATCCC |
| Il22ra2_F | GGAAATATTTGTTATTGGTGGAGTTTAA |
| Il22ra2_R | CTAACAACCAAAATTCCTTTCACTAAAA |
| Niacr1_F | TGAGTGGATTTGGTTAGTTTAGTTTTAGAG |
| Niacr1_R | ATTTCCTAATAACCATTCAATCATCTCTTT |
| Calm13_F | TAGTTTTTGTGGGGTTTTGATTTAG |
| Calm13_R | CCAAACTAACCACATCTACCACATC |
| Olfm4_F | GGTGTAAGGTGTGGATAGGATTTAG |
| Olfm4_R | AAACAAAACTAAAAAACCAAAAAAAA |
| Lgals2_F | GGAAGTGGGTGTAGAGGGTTAGTATT |
| Lgals2_R | TCAAAATAAATAAAAAACCATCTTCCTATT |
| Reg3b_F | GAGGGTGTGGATGGTTTAAATTTTAT |
| Reg3b_R | AATTCACCCTTACCTATCTTCCAAAA |
| Djc22_F | TTATAGTGTAAAATTGGGTGAGTTTGG |
| Djc22_R | TCACCTCCACACAAATAAACTTTATC |
| Tff3_F | AAGAAATTATGTATTTTTTTAGTGGTGATT |
| Tff3_R | AAATCCACAAAACCCAACAAAC |
| BsTBX3 | GAGTGGAGTAATGTTTGATATGTGGTGTAT |
| BsTBX3 | AAATCTAAATTTCCCCCTAACCTCCC |
| BsTCF4 | TATTTTATAAGAAGAGGGTGGGGTTG |
| BsTCF4 | CCCAAAAAATTACTTTCTAAATTCAAATTT |
| BsChr19 | TTAGAAAGTTAGGTGGGAATTGAAATG |
| BsChr19 | CAACAACAACCAACCAACCAAC |
| BsLRP1 | TTTATATTGGTTTTAGGGTTTTTGAAT |
| BsLRP1 | TTCAACAAACACACACTAAATATTTAACAC |
| Chr8bs | AGGTTATGTTTGGGATTTTGAATTTTTAG |
| Chr8bs | CAAACCAAAAACTCCCCTTACTCTC |
| Chr8bs_1 | TGTTTTTAGTGGGAGTGGTAGAAGGT |
| Chr8bs_1 | ACCATAAACACAAAAACTTCCCACAT |
| Chr8bs_2 | GTGATATTTTTGAGAATTTTGTTAGGTAAA |
| Chr8bs_2 | AAAATAACAAAAAAACAAACCTTCTTAC |

**Table S3 List of 4C primers used in this study.**

| >1_DpnII_187138555_187139504_DpnII_271 | AATGATACGGCGACCACCGAACACTCTTTCCCTACACGACGCTCTTCCGATCTCTACAGACTCTCCCGTGATC |
| --- | --- |
| >1_DpnII_187138555_187139504_BfaI_of_DpnII_271 | CAAGCAGAAGACGGCATACGATGAGTTCTGTAAGAGCCACA |
| >2_DpnII_167200835_167201272_DpnII_0 | AATGATACGGCGACCACCGAACACTCTTTCCCTACACGACGCTCTTCCGATCTcctccaaaggggttgagatc |
| >2_DpnII_167200835_167201272_Csp6I_of_DpnII_0 | CAAGCAGAAGACGGCATACGAACTCAAGTGCACACACACAA |
| >5_DpnII_33426765_33427733_DpnII_-1 | AATGATACGGCGACCACCGAACACTCTTTCCCTACACGACGCTCTTCCGATCTTGGTTTCTGGGCCTCTGATC |
| >5_DpnII_33426765_33427733_Csp6I_of_DpnII_-1 | CAAGCAGAAGACGGCATACGACTTAAGTGTTGACTGGAGCC |
| >5_DpnII_101070094_101070536_DpnII_0 | AATGATACGGCGACCACCGAACACTCTTTCCCTACACGACGCTCTTCCGATCTCATCACGGACACTACAGATC |
| >5_DpnII_101070094_101070536_BfaI_of_DpnII_0 | CAAGCAGAAGACGGCATACGATGACTTCTCTTAAGGTTGACG |
| >5_DpnII_119523945_119524925_DpnII_0 | AATGATACGGCGACCACCGAACACTCTTTCCCTACACGACGCTCTTCCGATCTTCATGTGCTCACACTTGATC |
| >5_DpnII_119523945_119524925_Csp6I_of_DpnII_0 | CAAGCAGAAGACGGCATACGAtggaagtggtgttacagaca |
| >6_DpnII_38852465_38853026_DpnII_0 | AATGATACGGCGACCACCGAACACTCTTTCCCTACACGACGCTCTTCCGATCTaaaagaaccaccacaTGATC |
| >6_DpnII_38852465_38853026_BfaI_of_DpnII_0 | CAAGCAGAAGACGGCATACGAcattcagcctttaagctcag |
| >6_DpnII_86604244_86605093_DpnII_0 | AATGATACGGCGACCACCGAACACTCTTTCCCTACACGACGCTCTTCCGATCTTCTCATGTGGCAGCAGGATC |
| >6_DpnII_86604244_86605093_Csp6I_of_DpnII_0 | CAAGCAGAAGACGGCATACGAGTAGTCTTTTCGTAGTGAGCG |
| >6_DpnII_147012895_147013570_DpnII_335 | AATGATACGGCGACCACCGAACACTCTTTCCCTACACGACGCTCTTCCGATCTGGGAAGGATAAAGAAAGATC |
| >6_DpnII_147012895_147013570_Csp6I_of_DpnII_335 | CAAGCAGAAGACGGCATACGATCCTCTTTCCTTCCTTAGGT |
| >7_DpnII_63166482_63167848_DpnII_0 | AATGATACGGCGACCACCGAACACTCTTTCCCTACACGACGCTCTTCCGATCTCACTGATGGCCTCTTGGATC |
| >7_DpnII_63166482_63167848_BfaI_of_DpnII_0 | CAAGCAGAAGACGGCATACGAGCAGCGTGGATACATACTATC |
| >8_DpnII_18792138_18792868_DpnII_776 | AATGATACGGCGACCACCGAACACTCTTTCCCTACACGACGCTCTTCCGATCTAGGATTCTGCAAGAGGGATC |
| >8_DpnII_18792138_18792868_Csp6I_of_DpnII_776 | CAAGCAGAAGACGGCATACGACCAGTTTGCAATCTTTCAAT |
| >8_DpnII_89289667_89291143_DpnII_0 | AATGATACGGCGACCACCGAACACTCTTTCCCTACACGACGCTCTTCCGATCTGTTGTAGTCGTGGCTGGATC |
| >8_DpnII_89289667_89291143_Csp6I_of_DpnII_0 | CAAGCAGAAGACGGCATACGATCTGTCTGTTGCCTAATCCT |
| >8_DpnII_129372325_129373075_DpnII_384 | AATGATACGGCGACCACCGAACACTCTTTCCCTACACGACGCTCTTCCGATCTCTAAGTGGAGGTCAGAGATC |
| >8_DpnII_129372325_129373075_Csp6I_of_DpnII_384 | CAAGCAGAAGACGGCATACGAGTTTCAGGAGGTGACAACAT |
| >9_DpnII_114625844_114626143_DpnII_154 | AATGATACGGCGACCACCGAACACTCTTTCCCTACACGACGCTCTTCCGATCTTGAGTGTTCAATGAGTGATC |
| >9_DpnII_114625844_114626143_Csp6I_of_DpnII_154 | CAAGCAGAAGACGGCATACGACCCTTCACAACAGTCTCTTC |
| >10_DpnII_127029769_127030549_DpnII_0 | AATGATACGGCGACCACCGAACACTCTTTCCCTACACGACGCTCTTCCGATCTtcactcctccataaatgatc |
| >10_DpnII_127029769_127030549_BfaI_of_DpnII_0 | CAAGCAGAAGACGGCATACGAAGAATGACCAGATACCAACG |
| >15_DpnII_72956875_72957262_DpnII_0 | AATGATACGGCGACCACCGAACACTCTTTCCCTACACGACGCTCTTCCGATCTgtctggtccctgtggagatc |
| >15DpnII__72956875_72957262_BfaI_of_DpnII_0 | CAAGCAGAAGACGGCATACGAGCATCAGAGCTACTCTTGCT |
| >17_DpnII_39148130_39148450_DpnII_3422 | AATGATACGGCGACCACCGAACACTCTTTCCCTACACGACGCTCTTCCGATCTtgtttttattcctggtgatc |
| >17_DpnII_39148130_39148450_BfaI_of_DpnII_3422 | CAAGCAGAAGACGGCATACGACTGGTTCATGAAGGTGTAGG |
| >18_DpnII_39199849_39200660_DpnII_877 | AATGATACGGCGACCACCGAACACTCTTTCCCTACACGACGCTCTTCCGATCTtattccctaggcaagagatc |
| >18_DpnII_39199849_39200660_Csp6I_of_DpnII_877 | CAAGCAGAAGACGGCATACGAACTCTCTGAGCAAGGGCT |
| >18_DpnII_74659111_74659796_DpnII_105 | AATGATACGGCGACCACCGAACACTCTTTCCCTACACGACGCTCTTCCGATCTGAAAATATTGGCGTCTGATC |
| >18_DpnII_74659111_74659796_BfaI_of_DpnII_105 | CAAGCAGAAGACGGCATACGATTAAATGATAAATCCACCCG |
| >18_DpnII_75602457_75603145_DpnII_-1 | AATGATACGGCGACCACCGAACACTCTTTCCCTACACGACGCTCTTCCGATCTctcttggtcagtggcagatc |
| >18_DpnII_75602457_75603145_BfaI_of_DpnII_-1 | CAAGCAGAAGACGGCATACGAagttaagtggcttgcagaag |
| >19_DpnII_32614271_32615619_DpnII_746 | AATGATACGGCGACCACCGAACACTCTTTCCCTACACGACGCTCTTCCGATCTGAGAGACTGGGAGGGTGATC |
| >19_DpnII_32614271_32615619_Csp6I_of_DpnII_746 | CAAGCAGAAGACGGCATACGAAACAGTGGAAAGACAGGAAA |
| >19_DpnII_36439117_36440062_DpnII_893 | AATGATACGGCGACCACCGAACACTCTTTCCCTACACGACGCTCTTCCGATCTTGAGGACTTTATCTCAGATC |
| >19_DpnII_36439117_36440062_BfaI_of_DpnII_893 | CAAGCAGAAGACGGCATACGACAGAAGGTTCTTAGCAGGAA |
| >19_5DpnII_5938072_55938514_DpnII_0 | AATGATACGGCGACCACCGAACACTCTTTCCCTACACGACGCTCTTCCGATCTCAATAGCGACTTCATGGATC |
| >19_DpnII_55938072_55938514_BfaI_of_DpnII_0 | CAAGCAGAAGACGGCATACGACCCATCCAAAGTTTACCTCT |

**Additional figure legends**

**Figure S1. Expression of marker genes in the purified cell populations.** Probe intensities from affimetrix micro-arrays of transcripts associated with different cell types or regions of the SI as indicated below the plot (TA=transit amplifying compartment).

**Figure S2. Mapping statistics.** (a) Read depth distribution of all CpGs. (b) General mapping statistics: including reads generated (paired-end) and mapped, average coverage, fraction of CpG covered, conversion efficiency and mean methylation. All mapped reads are required to map to a unique location. The column ‘uniquely mapped reads’ shows the number of reads after removing duplicate reads (with the same start and end positions).

**Figure S3. TSS methylation and gene expression in the SI.** (a,b,d) Genome browser views of Mash2, Olfm4 and 4930481A15Rik, respectively. (c) Expression levels of 4930481A15Rik within the SI as determined by microarray analysis. Y-axis reflects probe intensities.

**Figure S4. No changes in HMR distribution at transposons families or the expression of transposons.** (a) RT-qPCR analysis of transposon transcripts in the different cell types (Expression GFP_High was set to one). Technical triplicates are shown of one biological experiment. (b) Correlation of the amount of HMR in SINEs subdivided by transposon family between methylomes. (c) Correlation of the amount of HMR in LTRs subdivided by transposon family between methylomes. (d) Correlation of the amount of HMR in LINEs subdivided by transposon family between methylomes.

**Figure S5. Two examples of confirmation of DMRs called between the methylomes.** (a,b) Top: Genome browser view of Cyfip1 and Pdx-1 respectively, with black boxes indicating the CpGs tested. Left: Graph showing the methylation status of individual CpGs. Right: Bar diagram showing the relative expression in the different cell types (GFP_High was set to one). (c,d) Genome browser views of Epha2 and Ank3 respectively, with black boxes indicating the CpGs tested. These views correspond to Figures 3c and d. (e) Expression levels of the three DNMT genes throughout SI differentiation as determined by microarray analysis. Y-axis reflects probe intensities.

**Figure S6. Additional ChIP-qPCR and ChIP-Seq analysis.** (a) Chip-qPCR results for H3K9me3 performed on chromatin of villus epithelium for eight individual DMRs. DMRs are labelled after their closest gene (as indicated), or after their chromosomal location. * P<0.05 (student t-test). (b-d) Distribution of ChIP-Seq reads of H3K4me1, H3K27ac and H3K4me3 ChIPs over all DMRs.

**Figure S7. 4C domainograms of three intergenic DMRs**

(a-c) Domainograms of three interaction profiles of DMRs in which the DMR loops to the TSS of a gene. Significant interactions are indicated in the domainogram using different colours, which correspond to different P-values as indicated on the right site lower panel. Above the domainogram the normalized reads are plotted. The top of the figure indicates genes. The vertical arrows indicate the 4C interaction with a TSS. The significantly differentially expressed genes P<0.01 (student t-test) are colour coded as indicated on the right site upper panel. Below the figure the chromosomal location is shown. N4bp1 is not colour coded as its significance is P<0.05 (student t-test). (d-j) Genome browser view of short range interaction from intergenic (e) and intragenic DMRs (f-k). Where horizontal arrows indicate TSS and vertical arrows the DMR/viewpoint.

**Figure S8. A role for TCF4 in DMR formation.**

(a) Recombination efficiency of the p450-CRE three days post induction. (b-e) Different immune stainings to probe normal SI development. (B) Depicts Ki67 staining to show the presence of proliferating cells. (c-e) Stainings with Lys2, Decam and Synaptohpysin which stains the following three differentiated cell types paneth cell, neuroendocrine cell and Tuft cells (f-h) Left panel indicates tested CpGs of different DMRs after TCF4 ablation in the SI and the values found in the methylomes. Above the panels the genomic coordinates of the tested DMR is indicated and if applicable the gene it is located in or contacts by looping. Right panel shows ChIP-qPCR enrichments of TCF4 at the DMR tested in the panel to the left. (i) Expression levels of Tcf4 within the SI as determined by microarray analysis. Y-axis reflects relative probe intensities. Probe intensity was set to 1 in the Lgr5_High populations.
