# Supplementary material for: DNA methylation dynamics during intestinal stem cell differentiation reveals enhancers driving gene expression in the villus
Source: Genome Biol. 2013 May 28;14(5):R50. doi: 10.1186/gb-2013-14-5-r50 (PMC4053812; doi:10.1186/gb-2013-14-5-r50)

Kaaij et al. Additional Figure 1.

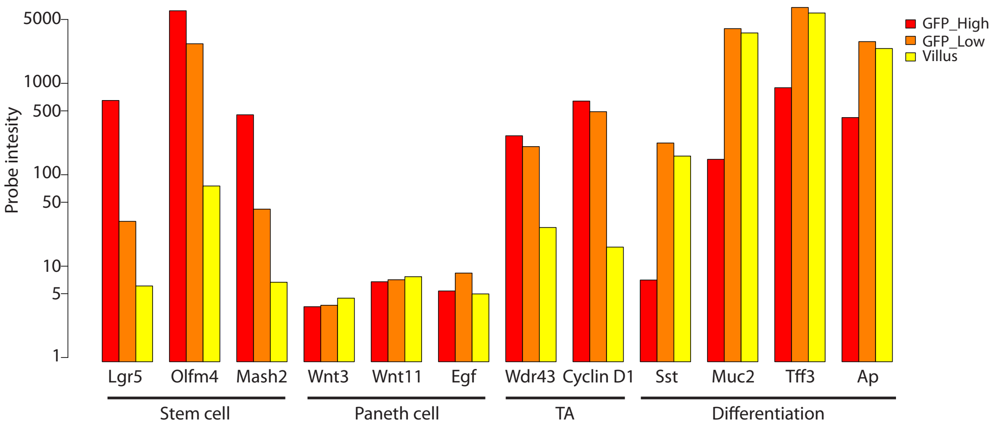

Kaaij et al. Additional Figure 2.

a

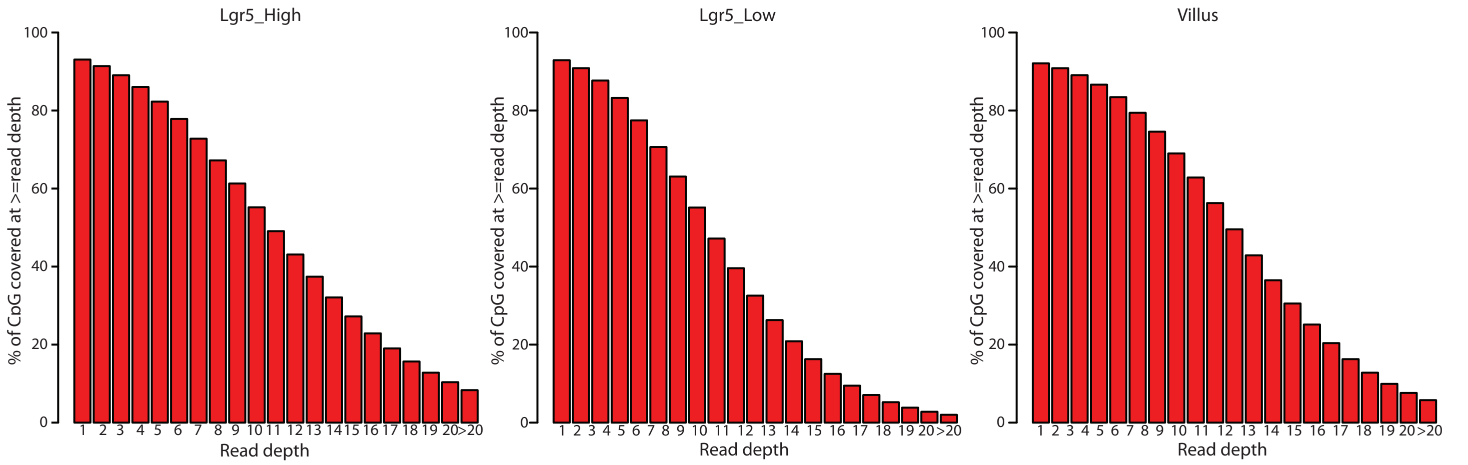

b

Cell type	Sequenced reads	Mapped Reads	Uniquely mapped Reads	Average coverage	CpGs covered	Conversion rate
GFP_High	1066345482	683786193	347258893	10.8	0.93	>0.99
GFP_Low	859216428	484006844	289100046	8.6	0.93	>0.99
Villus	848300040	657118702	347239683	10.6	0.92	>0.99

Kaaij et al. Additional Figure 3.

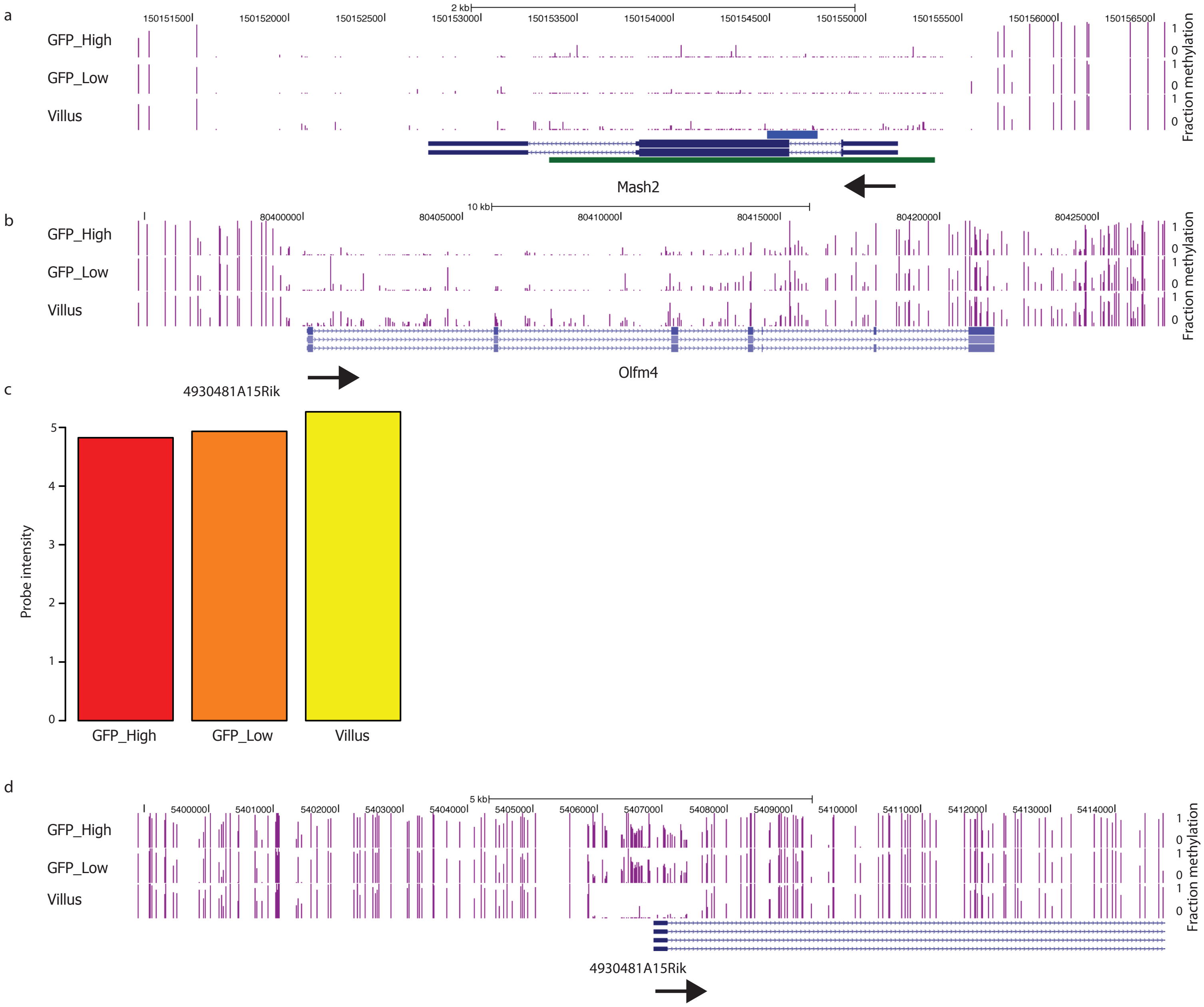

Kaaij et al. Additional Figure 4.

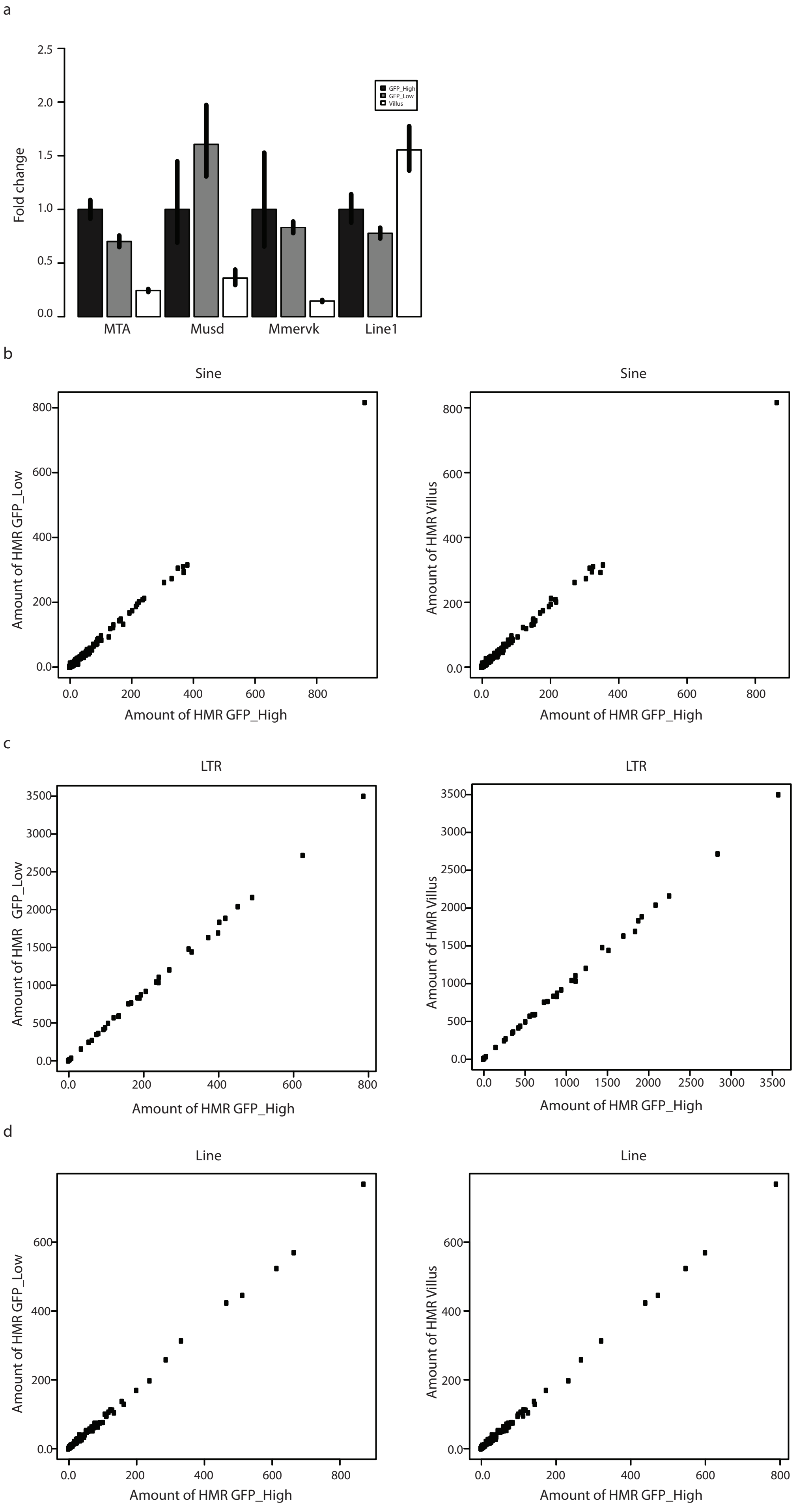

Kaaij et al. Additional Figure 5.

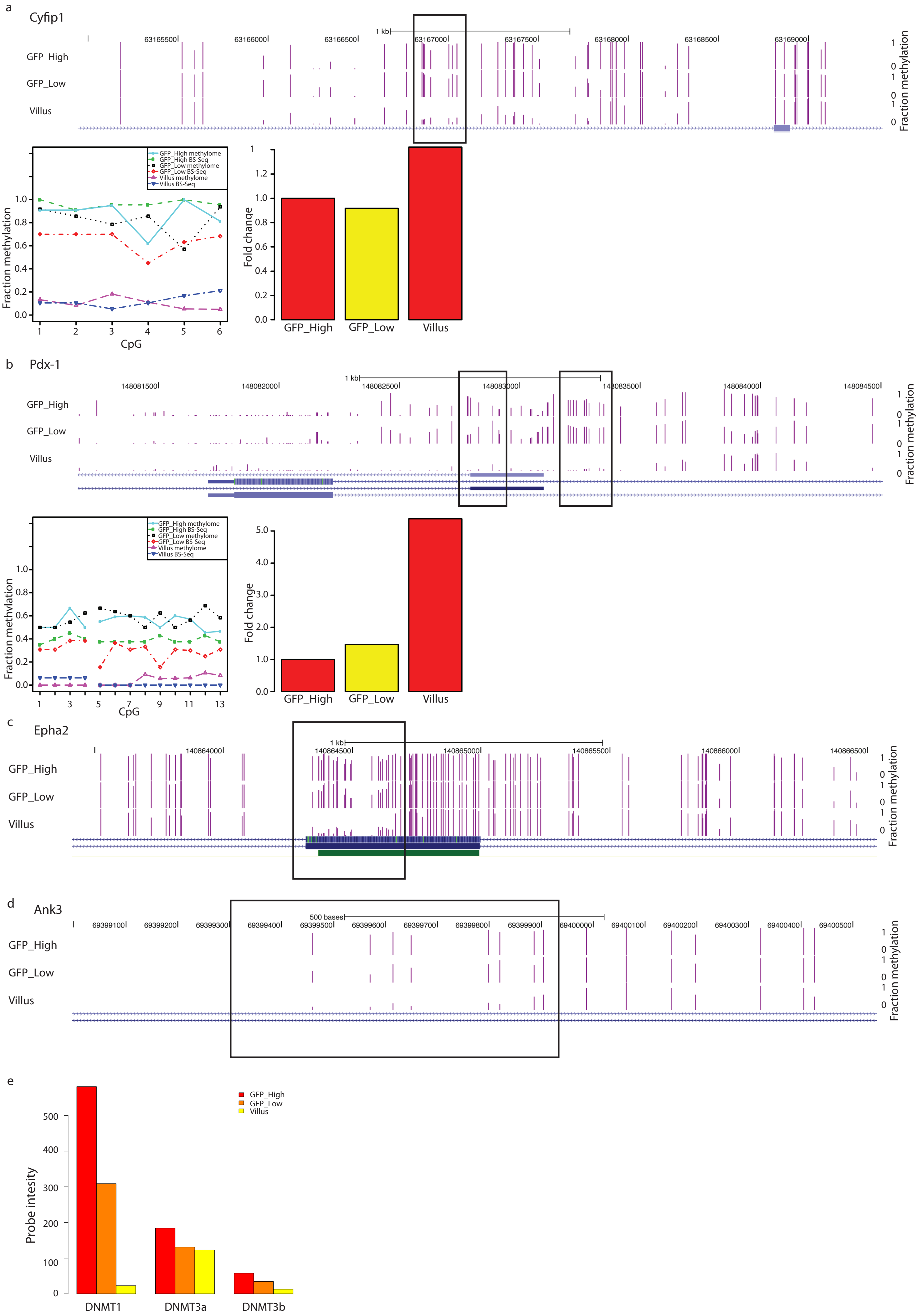

Kaaij et al. Additional Figure 6.

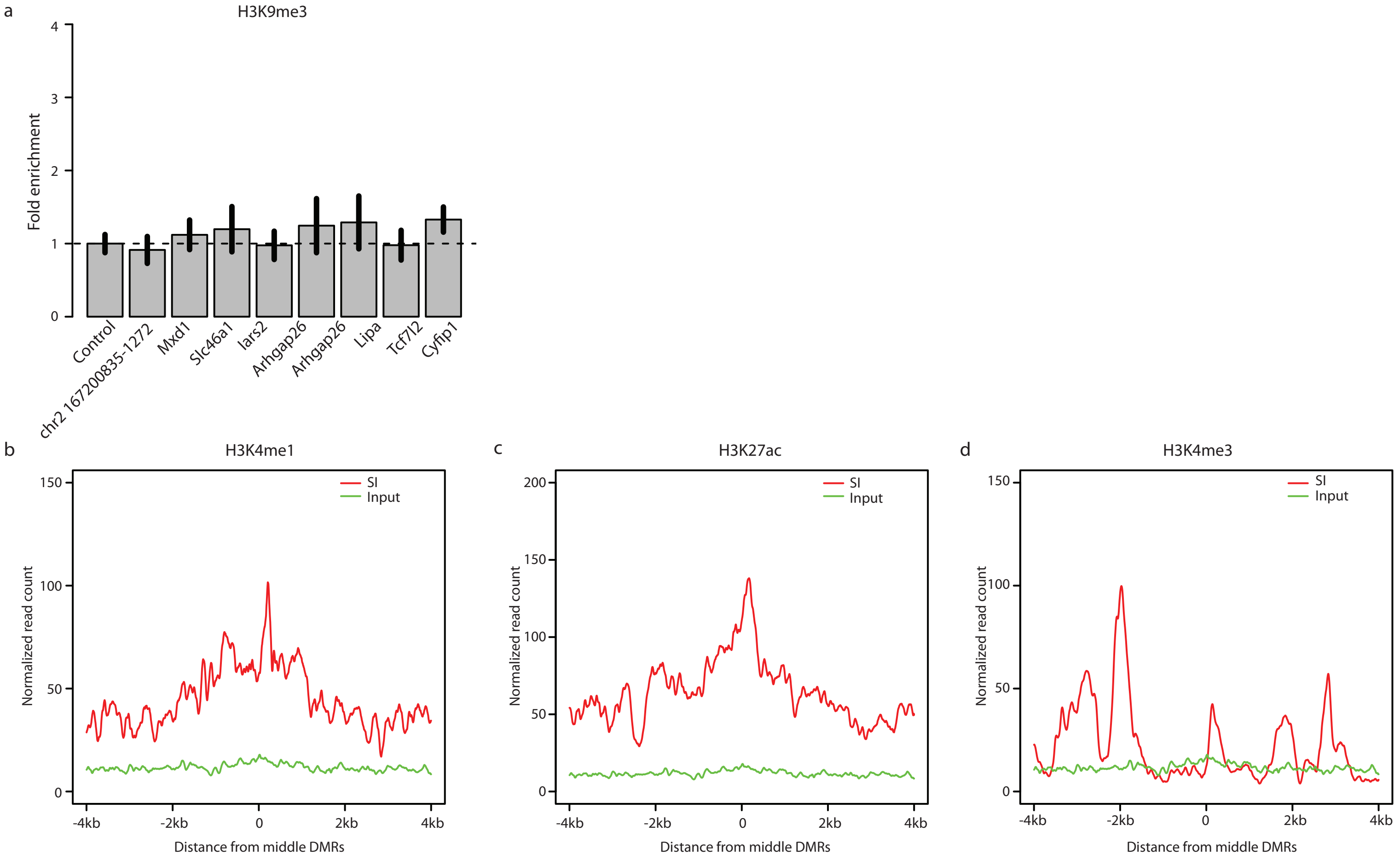

Kaaij et al. Additional Figure 7

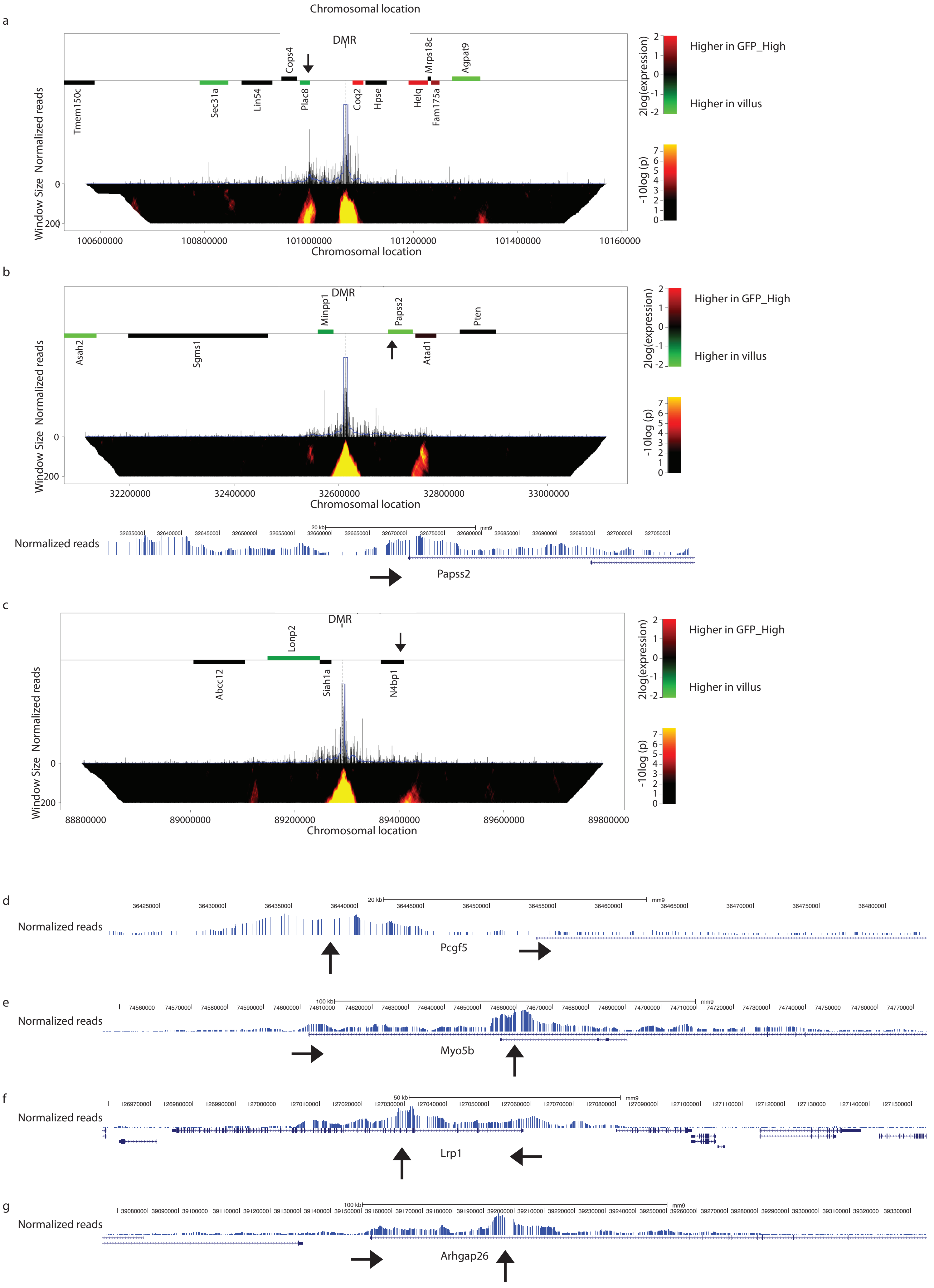

Kaaij et al. Supplemental Figure 7

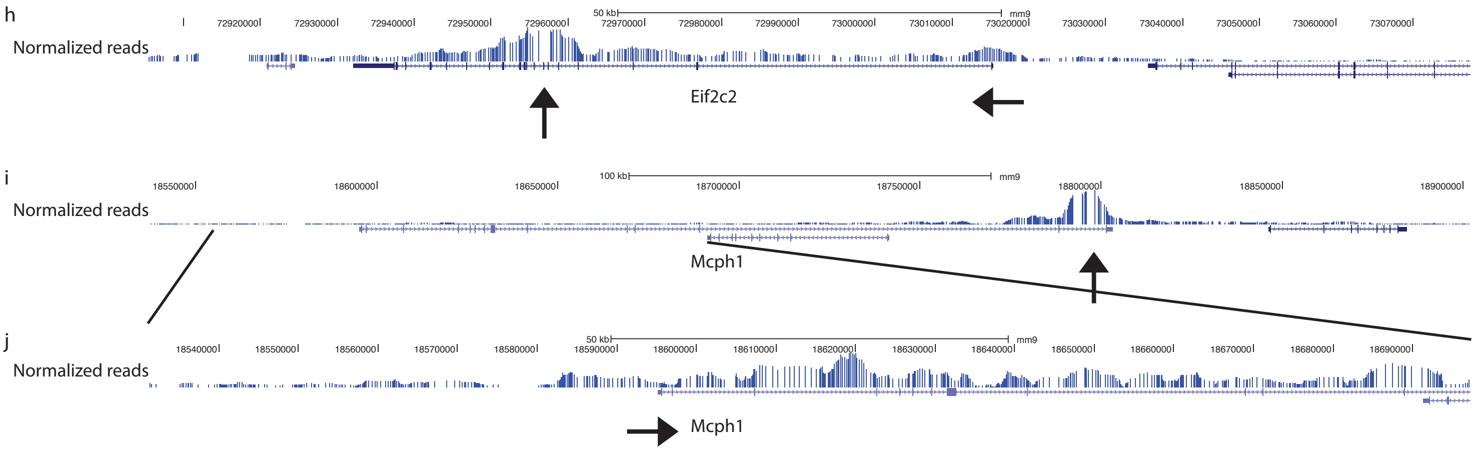

Kaaij et al. Additional Figure 8

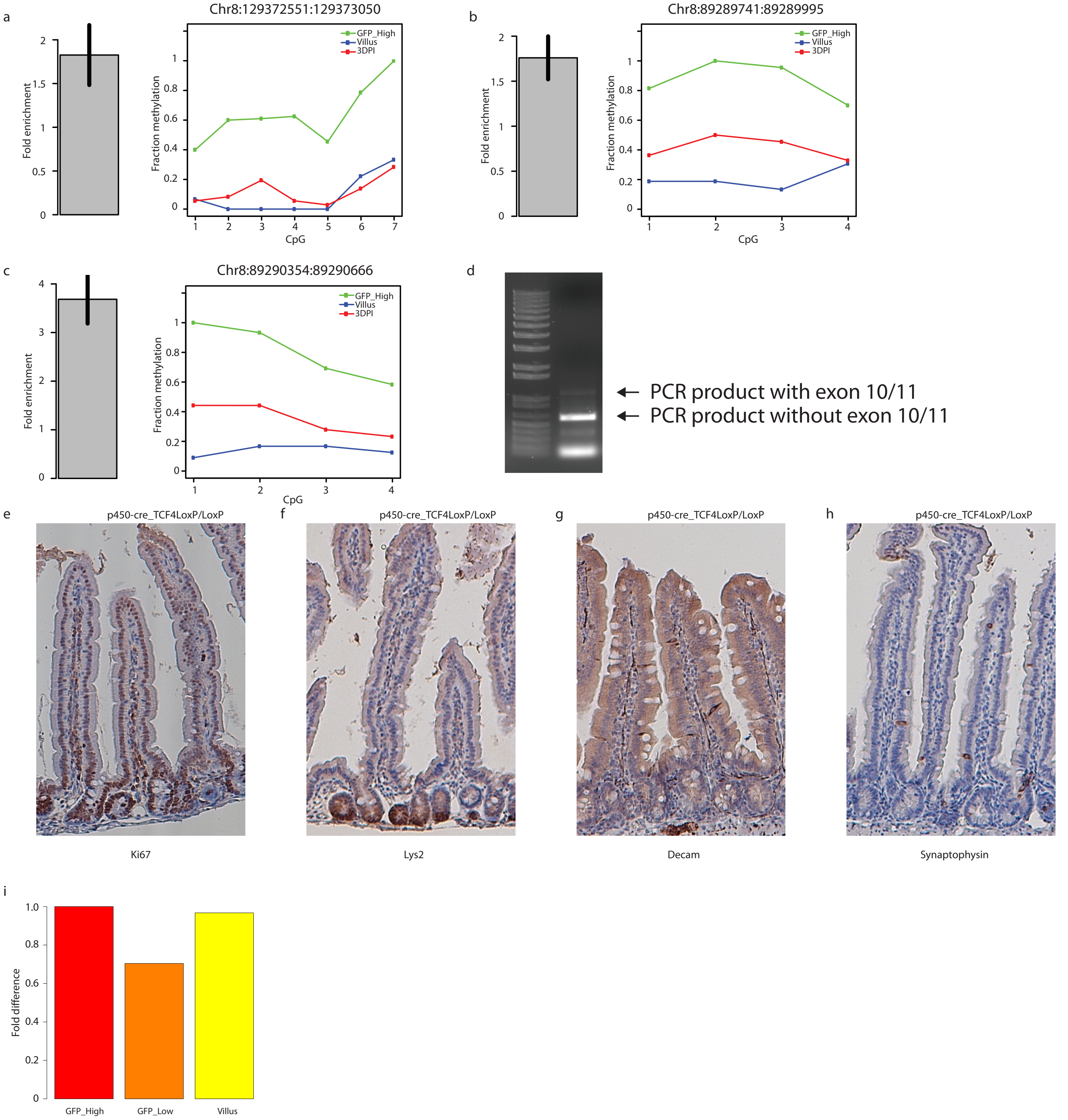

Supplement: Additional file 2 — Figure S1: Expression of marker genes in the purified cell populations. Expression of a set of marker genes, derived from Affimetrix micro-array experiments, in the three sorted cell populations. Figure S2: Mapping statistics. Statistics on the high-throughput sequencing described in this manuscript. Figure S3: TSS methylation and gene expression in the SI. Figure showing genome browser view of different genes. Figure S4: No changes in HMR distribution or expression of transposons. Figure showing the lack of HMR distribution at transposons. Figure S5: Two examples of confirmation of DMRs called between the methylomes. Manual BS-Seq conformation of two DMRs called in the methylomes. Figure S6: Additional ChIP-qPCR and ChIP-Seq analysis. Chip-qPCR and Chip-seq analysis of the DMRs called between SI stem cells and villus. Figure S7: 4C domainograms of three intergenic DMRs. Additional 4C experiments performed on DMRs called between SI stem cells and villus. Figure S8: A role for TCF4 in DMR formation. Additional data implication TCF4 in DMR formation in the SI. [file gb-2013-14-5-r50-S2.PDF]
